# SR9009 administered for one day after myocardial ischemia-reperfusion prevents heart failure in mice by targeting the cardiac inflammasome

**DOI:** 10.1038/s42003-019-0595-z

**Published:** 2019-10-03

**Authors:** Cristine J. Reitz, Faisal J. Alibhai, Tarak N. Khatua, Mina Rasouli, Byram W. Bridle, Thomas P. Burris, Tami A. Martino

**Affiliations:** 1 Centre for Cardiovascular Investigations, Department of Biomedical Sciences, University of Guelph, Guelph, Ontario N1G2W1 Canada; 2 Department of Pathobiology, University of Guelph, Guelph, Ontario N1G2W1 Canada; 3 Center for Clinical Pharmacology, Washington University School of Medicine and St. Louis College of Pharmacy, St. Louis, MO 63104 USA

**Keywords:** Heart failure, Cardiovascular diseases

## Abstract

Reperfusion of patients after myocardial infarction (heart attack) triggers cardiac inflammation that leads to infarct expansion and heart failure (HF). We previously showed that the circadian mechanism is a critical regulator of reperfusion injury. However, whether pharmacological targeting using circadian medicine limits reperfusion injury and protects against HF is unknown. Here, we show that short-term targeting of the circadian driver REV-ERB with SR9009 benefits long-term cardiac repair post-myocardial ischemia reperfusion in mice. Gain and loss of function studies demonstrate specificity of targeting REV-ERB in mice. Treatment for just one day abates the cardiac NLRP3 inflammasome, decreasing immunocyte recruitment, and thereby allowing the vulnerable infarct to heal. Therapy is given in vivo, after reperfusion, and promotes efficient repair. This study presents downregulation of the cardiac inflammasome in fibroblasts as a cellular target of SR9009, inviting more targeted therapeutic investigations in the future.

## Abstract (web summary)

Reitz et al. show that targeting the circadian regulator REV-ERB for just one day after myocardial ischemia-reperfusion benefits cardiac repair by reducing the NLRP3 inflammasome and immune recruitment to infarcted myocardium, and thus preventing the development of heart failure.

## Introduction

Ischemic heart disease leading to myocardial infarction (MI, heart attack) is a leading cause of morbidity and mortality^1^. Although many patients reach hospitals in a timely manner and undergo post-ischemia reperfusion (mI/R), unfortunately this triggers a profound inflammatory response termed “reperfusion injury”, which in turn leads to infarct expansion and adverse cardiac remodeling and pathological progression to heart failure (HF) for which there is no cure^2–5^. Although we cannot predict when an individual will experience an MI, we can devise strategies to reduce reperfusion injury after MI, thereby reducing deleterious remodeling to improve on quality of life and outcomes. Here, we demonstrate how targeting the circadian mechanism reduces the NLRP3 inflammasome, leading to less reperfusion injury. Treatment for as little as 1 day post-mI/R provides a window of time in which the infarct region can intrinsically heal, thereby reducing adverse remodeling and protecting against the development of HF.

The basis for our approach is built on the solid existing foundation of clinical and experimental evidence showing that the immune system plays a fundamental role in mitigating reperfusion injury post-mI/R^6–10^. It has long been thought that reducing the early inflammatory responses is a key promising strategy for intervention^4,9,11^. However, earlier studies targeting the immune system used broad approaches that failed to show clinical effectiveness, likely in large part because they mitigated not only the deleterious pathways involved in infarct healing but also the beneficial pathways that were crucial for healing^12^. Our innovative approach is based on specific targeting of the circadian mechanism, which we recently discovered is a key regulator of immunocyte recruitment to infarcted myocardium^10^. Here, we show that targeting the circadian mechanism at the time of reperfusion limits formation of the cardiac NLRP3 inflammasome to reduce the subsequent cascade of inflammatory responses that are involved in infarct expansion, with clear benefits on cardiac structure, function, and outcome.

Circadian medicine to benefit treatment of clinically relevant mI/R has never been pursued, because to date we lacked the pharmacology to do so. However, most recently we have developed pharmacology that targets the circadian mechanism, such as the core driver REV-ERB. In this study, we target REV-ERB post-mI/R to increase the constitutive repression of genes regulated by REV-ERB^13^, including the inflammasome that drives adverse immune recruitment to the infarcted myocardium. We use the clinically relevant murine coronary artery ligation/reperfusion mI/R model, and the REV-ERB agonist SR9009^13^, to simulate the possible effects and benefits on the evolution of ventricular remodeling in humans. Pharmacological targeting of the circadian mechanism can be given in vivo, after mI/R and alongside conventional therapies, and is highly effective for improving outcomes.

## Results

### Short-term in vivo SR9009 treatment post-reperfusion limits HF

**Fig. 1:**
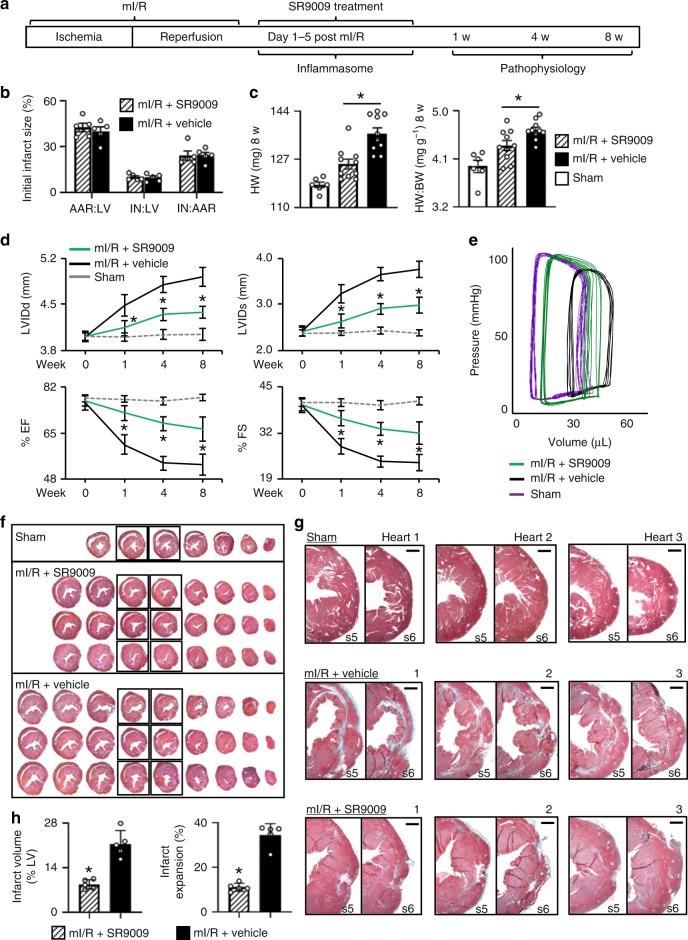
Short-term pharmacological targeting of REV-ERB after mI/R protects against HF. **a** Experimental design, left anterior descending coronary artery ligation for 45 min of ischemia followed by reperfusion and SR9009 treatment (i.p. once daily at ZT06 for up to 5 days), and pathophysiologic assessments up to 8 weeks post-mI/R. **b** All infarcts were reproducibly similar within the first day post-mI/R, as quantified by area at risk:left ventricle (AAR:LV), infarct area:LV (IN:LV), and infarct area:area at risk (IN:AAR) (*n* = 5 hearts/group). **c** mI/R mice treated with SR9009 for just 5 days had reduced heart weight (HW) and HW:body weight (BW) vs. mI/R+vehicle controls, at 8 weeks post-mI/R (see Table 1 for details). *p < 0.01 mI/R+SR9009 vs. mI/R+vehicle. **d** Functional benefits of short-term SR9009 treatment after mI/R include smaller LV diastolic (LVIDd) and systolic (LVIDs) dimensions and better % ejection fraction (% EF) and % fractional shortening (% FS), as compared with mI/R+vehicle, by echocardiography over 8 weeks post-mI/R (see Table 1 for all echocardiography values). *p < 0.0001 mI/R+SR9009 vs. mI/R+vehicle. **e** Preserved normal pressure–volume (PV) dynamics in SR9009-treated mI/R mice, as compared with mI/R+vehicle, representative image (see Table 1 for all hemodynamics values). **f** Representative hearts showing cardiac morphology in sham (top panel) and mI/R mice. The SR9009-treated mI/R hearts have less hypertrophy (less equidistant sections; middle panels), as compared with mI/R+vehicle mice (more equidistant sections; bottom panels). Heart sections are collected every 300 µm. Sections are stained with Masson’s trichrome. **g** The LV wall of sections #5 (s5) and #6 (s6) (from Fig. 1f) are shown with increased magnification to highlight comparisons in the infarct region. Top panel, normal LV wall in sham mice. Middle panel, transmural infarct in LV wall of mI/R hearts, as indicated by the blue-staining region. Bottom panel, animals treated short-term with SR9009 after mI/R have reduced infarct expansion by 8 weeks after mI/R. Scale bar (top right of each paired image) = 500 μm. **h** Quantification demonstrating that there is significantly (*p < 0.001) less infarct volume and infarct expansion in the SR9009-treated hearts by 8 weeks post-mI/R

We postulated that pharmacological targeting of REV-ERB after myocardial ischemia–reperfusion downregulates the NLRP3 inflammasome, leading to less reperfusion injury, reduced cardiac remodeling, and protects against heart failure (HF). To test this, we first used a murine model of the left anterior descending coronary artery ligation and reperfusion (mI/R), and treated with the REV-ERB agonist SR9009 for the first 5 days after mI/R, and followed pathophysiology outcomes to 8 weeks HF (Fig. 1a). Initial infarct size was consistent among mice, as expected, as measured by Evans Blue and tetrazolium chloride (TTC) staining (Fig. 1b). Importantly, even though all infarcts started the same, those in the SR9009-treated mI/R group developed significantly less (*p* < 0.01) cardiac hypertrophy versus those in the mI/R+vehicle group, as indicated by smaller heart weight (HW) and HW-to-body weight ratios (HW:BW) by 8 weeks HF (Fig. 1c). Thus these data suggest that short-term SR9009 treatment targeting REV-ERB after mI/R is protective and benefits long-term outcome.

To examine whether these treatment-dependent differences in cardiac enlargement post-mI/R correlate with in vivo structure and function changes, we performed echocardiography. Figure 1d and Table 1 show that the mI/R mice treated short-term with SR9009 developed significantly (*p* < 0.0001) less adverse remodeling with smaller left ventricular (LV) internal diastolic (LVIDd) and systolic (LVIDs) dimensions, along with better % ejection fraction (% EF) and % fractional shortening (% FS) by 8 weeks HF, as compared with vehicle-treated controls. Thus consistent with the heart size measurements, treatment with SR9009 for just the first few days after mI/R protects against adverse cardiac remodeling after reperfusion, and reduces progression to HF.

**Table 1:** Short-term treatment with the REV-ERB agonist SR9009 post-mI/R benefits long-term cardiac structure and function

	mI/R+SR9009	mI/R+vehicle	Sham
*n*	10	10	6
*Echocardiography baseline*			
LVIDd (mm)	4.02 ± 0.02	4.01 ± 0.02	4.02 ± 0.03
LVIDs (mm)	2.41 ± 0.03	2.43 ± 0.04	2.39 ± 0.03
EF (%)	76.81 ± 0.60	76.21 ± 0.87	77.76 ± 0.53
FS (%)	39.91 ± 0.53	39.75 ± 0.64	40.69 ± 0.50
HR (bpm)	472 ± 5	472 ± 4	468 ± 7
*1 week post-mI/R*			
LVIDd (mm)	4.14 ± 0.04*	4.49 ± 0.11	4.01 ± 0.03
LVIDs (mm)	2.65 ± 0.05*	3.23 ± 0.14	2.38 ± 0.02
EF (%)	72.31 ± 0.87*	60.75 ± 2.24	77.55 ± 0.49
FS (%)	36.15 ± 0.68*	28.22 ± 1.54	40.54 ± 0.44
HR (bpm)	455 ± 7	460 ± 8	474 ± 7
*4 weeks post-mI/R*			
LVIDd (mm)	4.35 ± 0.03*	4.80 ± 0.09	4.05 ± 0.03
LVIDs (mm)	2.90 ± 0.04*	3.65 ± 0.10	2.43 ± 0.03
EF (%)	68.37 ± 0.82*	53.84 ± 1.44	76.78 ± 0.58
FS (%)	33.14 ± 0.57*	23.92 ± 0.79	39.81 ± 0.53
HR (bpm)	468 ± 4	469 ± 5	485 ± 5
*8 weeks post-mI/R*			
LVIDd (mm)	4.38 ± 0.04*	4.92 ± 0.11	4.05 ± 0.04
LVIDs (mm)	2.99 ± 0.07*	3.77 ± 0.12	2.39 ± 0.03
EF (%)	66.44 ± 1.67*	53.31 ± 1.46	78.10 ± 0.48
FS (%)	31.93 ± 1.15*	23.61 ± 0.84	40.99 ± 0.45
HR (bpm)	461 ± 9	449 ± 4	471 ± 3
*Hemodynamics and morphometry 8 weeks post-mI/R*			
LVESP (mmHg)	97.11 ± 1.29*	91.43 ± 1.03	98.09 ± 2.14
LVEDP (mmHg)	1.66 ± 0.84	1.83 ± 0.62	0.04 ± 0.55
LVESV (μl)	17.83 ± 1.15*	28.58 ± 1.70	10.85 ± 0.68
LVEDV (μl)	42.02 ± 1.04*	48.90 ± 1.30	37.19 ± 0.96
SV (μl)	24.19 ± 0.68*	20.32 ± 0.79	26.34 ± 0.64
CO (mL min^−1^)	12.16 ± 0.47*	9.97 ± 0.32	13.33 ± 0.64
+dP/dt_max_ (mmHg s^−1^)	9302 ± 339*	7275 ± 358	10093 ± 819
−dP/dt_min_ (mmHg s^−1^)	−8543 ± 223*	−6621 ± 374	−9262 ± 517
SBP (mmHg)	95.87 ± 0.86*	91.12 ± 0.66	97.76 ± 1.24
DBP (mmHg)	58.67 ± 0.79	57.28 ± 1.31	61.93 ± 1.40
MAP (mmHg)	70.34 ± 0.61	67.88 ± 0.95	72.91 ± 1.15
HW (mg)	125.30 ± 1.77*	136.10 ± 2.03	118.00 ± 1.00
HW:BW (mg g^−1^)	4.35 ± 0.09*	4.64 ± 0.06	3.96 ± 0.12
BW (g)	28.89 ± 0.64	29.37 ± 0.48	30.02 ± 1.01
HR (bpm)	503 ± 13	495 ± 22	506 ± 18

*LVIDd* left ventricle internal dimensions at diastole, *LVIDs* LV internal dimensions at systole, *% EF* % ejection fraction, *% FS* % fractional shortening, HR heart rate, *LVESP* left ventricle (LV) end systolic pressure, *LVEDP* LV end diastolic pressure, *LVESV* LV end systolic volume, *LVEDV* LV end diastolic volume, *SV* stroke volume, *CO* cardiac output, *dP/dt*_*max*_ *and dP/dt*_*min*_ maximum and minimum first derivative of LV pressure, *SBP* systolic blood pressure, *DBP* diastolic blood pressure, *MAP* mean arterial pressure, *HW* heart weight, *BW* body weight

*n*  =  8 mI/R+SR9009, mI/R+vehicle, *n * =  6 sham for hemodynamics. *p < 0.05 mI/R+SR9009 vs. mI/R+vehicle, by Student’s *t* test. Values are mean  ±  SEM

To establish whether these functional improvements in the SR9009-treated group were also associated with better cardiac hemodynamic responses, we next measured these in vivo. The SR9009-treated mI/R mice had significantly (*p* < 0.005) better LV end systolic pressure (LVESP) and end systolic (LVESV) and diastolic (LVEDV) volumes compared with the vehicle-treated controls (Table 1, Fig. 1e). Consistent with the better cardiac hemodynamics, these mice also exhibited better (*p* < 0.005) stroke volume, cardiac output, dP/dt_max_ and dP/dt_min_ values, and blood pressure as compared with vehicle-treated controls (Table 1). Thus short-term treatment with SR9009 results in better cardiac function and reduced progression to HF as compared with mice not given the drug in the early days post-mI/R.

In view of the better cardiac function in the SR9009-treated mice, we next investigated whether this was associated with reduced cardiac remodeling and less infarct expansion. Histopathology revealed that SR9009-treated mice developed less cardiac hypertrophy (fewer equidistant sections) by 8 weeks HF versus vehicle-treated controls (Fig. 1f). Moreover, the treatment clearly prevented consequent damage to the LV infarct region, versus controls. As shown in Fig. 1g, a normal LV wall in mice is comprised of healthy myocardium (top panels), whereas the LV wall in the mI/R + vehicle mice exhibited clear transmural infarcts by 8 weeks HF (middle panels). In contrast, the LV infarct expansion was greatly limited in the SR9009-treated mI/R mice (lower panel); indeed, the myocardium appeared almost as healthy as that of shams with no infarct at all. Moreover, the SR9009-treated group had significantly (*p* < 0.001) smaller infarct volumes and reduced infarct expansion as compared with the mI/R + vehicle group at 8 weeks after mI/R, and consistent with the improved outcomes (Fig. 1h). Thus collectively these functional and structural data demonstrate clear protection and that SR9009 given for just the first few days after reperfusion leads to reduced infarct expansion, better outcomes, and limits progression to HF.

### Pharmacological targeting of REV-ERB in the healing heart

**Fig. 2:**
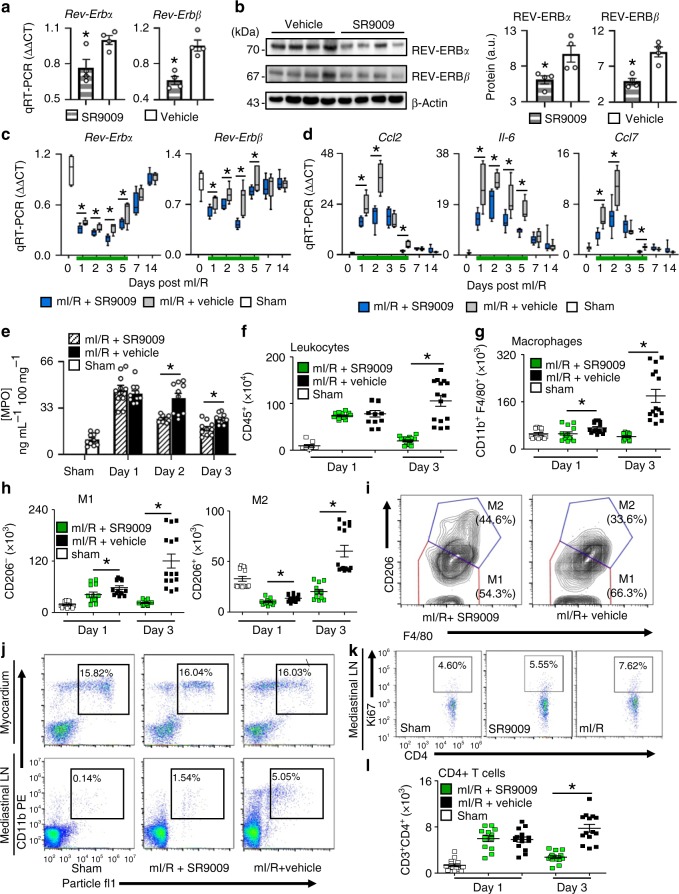
Short-term pharmacological targeting of REV-ERB benefits outcome post-mI/R by limiting inflammatory responses in infarcted myocardium. **a** SR9009 reduced cardiac Rev-Erbα and Rev-Erbβ mRNA expression and **b** protein abundance by sodium dodecyl sulfate polyacrylamide gel electrophoresis (SDS-PAGE) and western blot as quantified in vivo, ZT07, *p < 0.05 (*n* = 4 hearts/group). **c** SR9009 treatment for 5 days (green line) post-mI/R reduced Rev-Erbα and Rev-Erbβ mRNA expression in infarcted hearts only during the time of treatment, and **d** cardiac cytokine mRNA expression was also reduced in infarcted hearts, *p < 0.05 (*n* = 5 hearts/time point/group), see Supplementary Table 1 for RT-PCR values. **e** Neutrophil recruitment to the infarcted myocardium was reduced with SR9009 treatment vs. mI/R + vehicle by myeloperoxidase (MPO) assay (*p < 0.01, *n* = 5 hearts/time point/group). **f** Fewer leukocytes infiltrated infarcted myocardium following SR9009 treatment, as shown by flow-cytometry staining of CD45^+^ cells, and **g** less macrophages (CD11b^+^F4/80^+^) infiltrated, including both the (**h**) M1 (CD206^−^) and M2 (CD206^+^) subpopulations as compared with mI/R + vehicle controls, and **i** the proportion of reparative M2 cells over the adverse M1 macrophages was higher in SR9009-treated mice consistent with the improved outcomes (*p < 0.001, *n* = 5 hearts/time point/group). See Supplementary Fig. 1 for leukocyte gating, and Supplementary Table 2 for leukocyte values. **j** To assess T cell responses, we first injected fluorescent particles into the anterior free LV wall at the time of surgery, which are taken up by CD11b^+^ myeloid cells, and trafficked to the mediastinal lymph nodes (Supplementary Fig. 2). SR9009-treated mice had reduced trafficking to the heart-draining mediastinal lymph nodes by day 1, **k** resulting in less proliferation of Ki67^+^ lymphatic CD4^+^ T cells, and **l** reduced adverse T cell infiltration in the infarcted myocardium by day 3 (*p < 0.001, *n* ≥ 4 hearts/group)

To investigate the early mechanisms responsible, we first showed that SR9009 treatment downregulates *Rev-Erb* in the heart. We found that SR9009 treatment of normal mice decreased (*p* < 0.05) Rev-Erbα and Rev-Erbβ mRNA (Fig. 2a) and protein (Fig. 2b) expression in the heart, as compared with vehicle-treated controls. Moreover, we found that SR9009 treatment post-mI/R also decreased (*p* < 0.05) expression of both Rev-Erbα and Rev-Erbβ in the infarcted heart over all 5 days of treatment (Fig. 2c), consistent with the notion that the drug targets the circadian mechanism to improve outcomes (Fig. 2a–c; Supplementary Table 1). Next, since the circadian mechanism plays a role in modulating immune responses in the infarcted myocardium, we examined whether treatment with SR9009 also led to decreased inflammatory cytokine mRNA responses in the infarcted heart. As early as 1 day post-mI/R, cytokine mRNA levels were less (*p* < 0.05) in the SR9009-treated hearts, with less expression of monocyte chemoattractant protein 1 (Mcp1)/chemokine (C–C motif) ligand 2 [Ccl2] mRNA, (*p* < 0.05) interleukin 6 (Il-6) mRNA, and (*p* < 0.05) Ccl7 mRNA as compared with vehicle-treated mI/R controls (Fig. 2d). Moreover, there was less cytokine mRNA expression in the hearts of SR9009-treated mice for all 5 days of treatment compared with vehicle-treated controls, covering the critical period of reperfusion injury (Fig. 2d; Supplementary Table 1).

### SR9009 post-reperfusion reduces inflammatory responses in the heart

Immune responses in the first few days post-mI/R trigger infarct expansion. In order to determine the effect of SR9009 on mitigating these responses, we looked at inflammatory cell infiltration to the heart in the early days post-mI/R. We found less immunocyte recruitment following treatment with SR9009. We first investigated cardiac neutrophil recruitment by myeloperoxidase (MPO) activity; MPO is carried by neutrophil cells and thus serves as a biochemical marker of infiltration. Figure 2e shows that, compared with controls, the SR9009-treated mice had an attenuated response with less (*p* < 0.01) MPO activity in the heart at day 2 (25.17 ± 1.03 versus 39.96 ± 4.94 ng per mL per 100 mg tissue) and day 3 (18.46 ± 2.20 versus 25.23 ± 1.62 ng per mL per 100 mg tissue), further supporting the notion of reduced myocardial neutrophil recruitment in the SR9009-treated mice. Consistent with MPO as a surrogate marker of cellular reperfusion responses in LV remodeling post-mI/R, the sham mice had only minimal levels of activity in the heart (Fig. 2e). Thus these data show that targeting the circadian mechanism decreases inflammatory post-mI/R cellular responses to reduce infarct expansion.

To provide a more robust and definitive assessment of post-reperfusion cardiac inflammation, we next measured the total leukocyte, macrophage, and T cell infiltration in the mI/R myocardium by flow cytometry (Fig. 2; Supplementary Table 2, Supplementary Fig. 1). Overall, fewer (*p* < 0.001) leukocytes (CD45^+^) infiltrated the infarcted myocardium at day 3 post-mI/R with SR9009 treatment (Fig. 2f), consistent with downregulation of the inflammatory reperfusion response. We next examined the macrophage response in mI/R+SR9009 hearts, by flow cytometry. As shown in Fig. 2g, treatment led to significantly (*p* < 0.001) less macrophage infiltration (CD11b^+^F4/80^+^) to the infarcted myocardium for the first 3 days post-mI/R. Importantly, there were fewer (*p* < 0.001) cells of both the M1 (CD206^-^) and M2 (CD206^+^) subpopulation types following treatment with SR9009 (Fig. 2h). Moreover, there was also a higher proportion of reparative M2 type macrophages following SR9009 treatment, as compared with more adverse inflammatory M1 types in the untreated controls (Fig. 2i).

We also investigated adaptive (T cell) responses following treatment. We injected fluorescent particles into the LV wall at the time of surgery, and tracked outcomes using flow cytometry. We found that although the same proportion of myeloid cells (CD11b^+^) within the heart took up the particles, there was reduced trafficking of particle-positive myeloid cells to the heart-draining mediastinal lymph nodes of SR9009-treated mice as compared with the lymph nodes from mI/R + vehicle controls (Fig. 2j). Since T cells are activated in the mediastinal lymph nodes (Supplementary Fig. 2), this suggests the possibility of less overall activation of T cells following SR9009 treatment in vivo. Consistent with this notion, we found significantly (*p* < 0.001) less proliferation of T cells (CD3^+^CD4^+^Ki67^+^) in the mediastinal lymph node (Fig. 2k), and ultimately significantly (*p* < 0.001) less T cell infiltration in infarcted myocardium by day 3 (Fig. 2l; Supplementary Table 2). Taken together, our findings suggest that pharmacological targeting of the circadian factor REV-ERB for just the first few days after mI/R decreases both the innate and the subsequent adaptive inflammatory responses in the infarcted heart post-mI/R, and that this reduction leads to reduced infarct expansion and better outcomes.

### SR9009 post-reperfusion reduces the adverse NLRP3 inflammasome

**Fig. 3:**
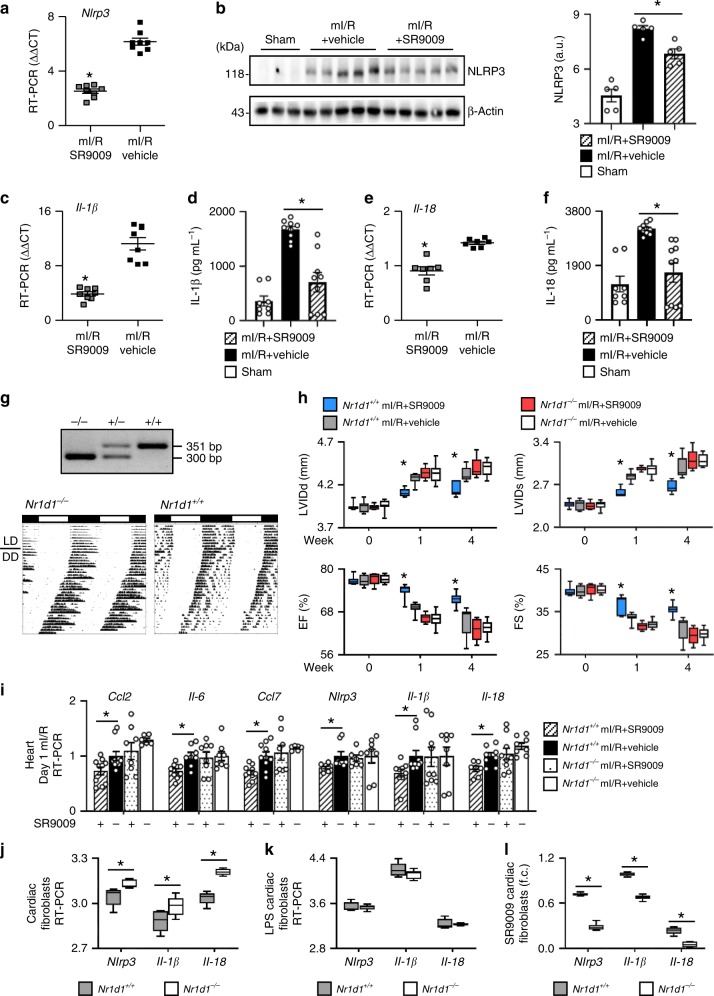
SR9009 reduces the adverse NLRP3 inflammasome, and genetic loss of REV-ERB repressor activity. **a** SR9009 treatment reduced cardiac Nlrp3 mRNA expression, and **b** protein abundance as early as 1 day post-mI/R as compared with vehicle-treated controls, *p < 0.01. SR9009 treatment also reduced inflammasome cytokines in the heart, including **c** Il-1β mRNA expression and **d** IL-1β protein, and **e** Il-18 mRNA expression and **f** IL-18 protein abundance versus controls, ZT07, *p < 0.01 (*n* = 5 hearts/group). **g** Representative genotyping of homozygous *Nr1d1*^*−/−*^, heterozygous *Nr1d1*^*+/−*^, and WT *Nr1d1*^*+/+*^ mice (top), and wheel running actigraphy (bottom) of mice under diurnal (12 h light:12 h dark; LD) and circadian (constant darkness; DD) conditions. *Nr1d1*^*−/−*^ mice exhibited a shortened free-running circadian period, as anticipated. **h** Homozygous (*Nr1d1*^*−/−*^) mice lacking REV-ERB were not protected by treatment with the REV-ERB agonist SR9009, with no benefits on cardiac structure and function post-mI/R as compared with WT littermates with a functional target, see Supplementary Table 3 for pathophysiology values. **i** Treatment with SR9009 also showed no benefits on the hearts of *Nr1d1*^−/−^ mice for inflammatory cytokine mRNA expression nor NLRP3 inflammasome expression as compared with WT littermates (ΔΔCT, *p < 0.05, *n* = 4 hearts/group). **j** *Nr1d1*^*−/−*^ cardiac fibroblasts in culture exhibited increased expression of Nlrp3, Il-1β, and Il-18 (inflammasome) mRNA levels, consistent with a loss of REV-ERB repressor activity, and **k** LPS stimulated the inflammasome in cardiac fibroblasts isolated from *Nr1d1*^*+/+*^ or *Nr1d1*^*−/−*^ mice, and **l** SR9009 was most effective at reducing the inflammasome activation in the *Nr1d1*^*+/+*^ cells with a functional REV-ERB target (1/CT, *p < 0.05, *n* = 4/group). See Supplementary Tables 3, 4, 5, and Supplementary Fig. 3 for genetic (*Nr1d1*, *Clock*^Δ19/Δ19^) loss and gain of function studies

The adverse NLRP3 inflammasome is integral to mI/R injury; it exacerbates myocardial damage. We postulated that REV-ERB activation with SR9009 would decrease the production of its molecular output NLRP3, which would contribute to the improved outcomes. Consistent with this notion, we show that SR9009-treated mice have decreased (*p* < 0.01) expression of the REV-ERB molecular output Nlrp3 mRNA (Fig. 3a) and protein (Fig. 3b) expression in the heart as early as 1 day post-mI/R. Moreover, we also observed decreased (*p* < 0.01) production of the REV-ERB molecular outputs Il-1β mRNA (Fig. 3c) and protein (Fig. 3d), and Il-18 mRNA (Fig. 3e) and protein (Fig. 3f), which along with NLRP3 contribute to inflammasome formation. Thus, at as early as 1 day post-mI/R, SR9009 treatment limits the induction of the adverse NLRP3 inflammasome, reducing myocardial damage.

### Genetic circadian models of loss and gain of function

SR9009 enhances REV-ERB repressor activity and this provides a mechanism through which SR9009 protects against adverse remodeling post-mI/R, as shown above. Conversely then, one would anticipate that loss of SR9009’s target REV-ERB would abolish the protective benefits of SR9009 treatment. To explore this, we used REV-ERB (*Nr1d1*^*−/−*^) mice. Genotyping and running wheel actigraphy are shown in Fig. 3g. The *Nr1d1*^*−/−*^ mice were subjected to mI/R, treated with SR9009, and pathophysiology measured. The *Nr1d1*^*−/−*^ mice were susceptible to HF, with increased LVIDd and LVIDs and decreased % EF and % FS by 4 weeks post-mI/R; they develop HF similar to WT mI/R littermates (Fig. 3h; Supplementary Table 3). As anticipated, we found no protective benefits of SR9009 in the *Nr1d1*^*−/−*^ mice lacking the REV-ERB target. Indeed, the *Nr1d1*^*−/−*^ mI/R mice treated with SR9009 were not different than the *Nr1d1*^*−/−*^ mI/R vehicle controls, or the WT-mI/R littermate controls, consistent with the notion that a functional REV-ERB target is necessary for SR9009 efficacy (Fig. 3h; Supplementary Table 3). Moreover, SR9009 treatment only attenuated (*p* < 0.05) cytokine and inflammasome mRNA expression in WT mice with a REV-ERB target, but not in the *Nr1d1*^*−/−*^ mice lacking the REV-ERB target (Fig. 3i). Similarly, loss of REV-ERB led to increased expression of Nlrp3, Il-1β, and Il-18 in *Nr1d1*^*−/−*^ cardiac fibroblasts in vitro, consistent with a loss of repressor activity (Fig. 3j). Moreover, while lipopolysaccharide (LPS) similarly stimulated the inflammasome in in vitro cardiac fibroblasts derived from WT or *Nr1d1*^*−/−*^ knockout hearts (Fig. 3k), SR9009 was more effective at reducing the inflammasome expression in WTs as compared with *Nr1d1*^*−/−*^ cells lacking the REV-ERB target (Fig. 3l). As a corollary to the genetic loss-of-function studies using the Nr1d1 mice, we also studied mI/R responses using *Clock*^Δ19/Δ19^ mice, as a model of genetic gain of REV-ERB repressor activity. *Clock*^Δ19/Δ19^ mI/R mice showed better cardiac structure and function as compared with WT mI/R controls, with comparable benefit to that observed in SR9009-treated mI/R mice (Supplementary Fig. 3, Supplementary Table 4). Moreover, *Clock*^Δ19/Δ19^ mI/R mice also showed less infiltration of leukocytes (CD45^+^), macrophages (CD11b^+^F4/80^+^), and T cells (CD4^+^) to the infarcted myocardium as compared with hearts from WT mI/R mice (Supplementary Fig. 3, Supplementary Table 5), consistent with REV-ERB activation. Thus collectively these data are consistent with the notion that targeting the circadian mechanism is sufficient to reduce inflammation in the infarcted myocardium, mediated by downregulation of the inflammasome sensor in the heart.

### Identifying cellular targets of SR9009 treatment post-mI/R

**Fig. 4:**
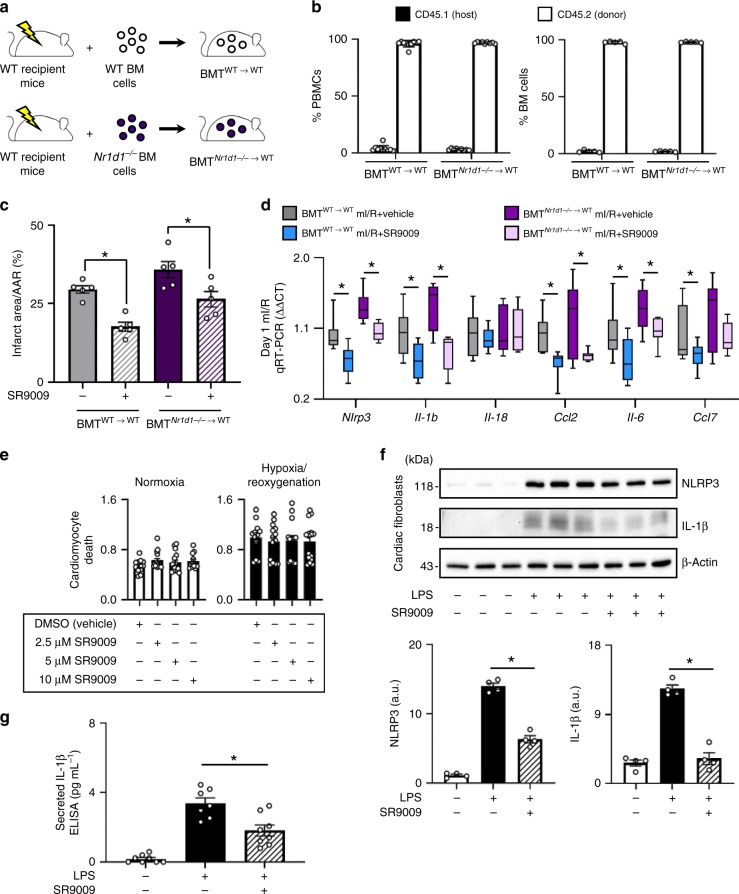
Cellular benefits of SR9009 treatment post-mI/R. **a** To investigate the role of immune cells, lethally irradiated WT (CD45.1^+^) mice were reconstituted with bone marrow cells from WT or *Nr1d1*^*−/−*^ donor (CD45.2^+^) mice, generating bone marrow transplant BMT^WT→WT^ or BMT^Nr1d1*−/−*→WT^ chimeras. **b** Peripheral blood mononuclear cells (PBMCs) and bone marrow cells had >96% reconstitution with CD45.2^+^ donor cells, at 8 weeks after BMT by flow cytometry (*n* ≥ 5/group). BMT mice were subjected to mI/R and treatment with SR9009, and **c** infarct size by day 2 was significantly reduced, and **d** there was less activation of cardiac inflammasome and cytokine mRNA expression versus vehicle-treated controls, ZT07, *p < 0.05 (*n* = 5 hearts/group). **e** Cardiomyocytes treated with SR9009 showed no difference in hypoxia-induced cell death versus vehicle-treated controls (*n* = 6/group). **f** LPS activates the inflammasome in cardiac fibroblasts, and co-treatment with SR9009 decreased levels in the cells, and **g** supernatant, *p < 0.05 (*n* = 4/group)

We investigated which cellular targets mediate the protective benefits of SR9009: immune cells, cardiac myocytes, or cardiac fibroblasts. First, we investigated the immune cells, by performing adoptive transfer experiments using *Nr1d1*^*−/−*^ mice to generate bone marrow transplant (BMT) chimeric mice. The experimental design to generate these mice (BMT^WT→WT^ and BMT^Nr1d1*−/−*→WT^) is illustrated in Fig. 4a. To verify the reconstitution of bone marrow after transplantation, we validated the presence of CD45.2^+^ donor cells in the blood and bone marrow of the BMT mice (Fig. 4b). Flow-cytometry analysis showed that peripheral blood and bone marrow cells consisted of > 96% donor cells by 8 weeks after BMT. These mice were then subjected to mI/R, treated with SR9009 or vehicle, and initial infarct size (Fig. 4c) and inflammasome and cytokine expression (Fig. 4d) were determined. We found that SR9009 works in WT mice transplanted with *Nr1d1*^*−/−*^ bone marrow, similar to the WT control mice, suggesting that immune cells in this context are not the primary protective targets of SR9009 treatment. Second, we investigated the cardiac myocytes as cellular targets, by performing viability assays in vitro under normal and hypoxia/reoxygenation conditions. We found that SR9009 treatment did not significantly rescue cardiomyocyte cell death under any conditions (Fig. 4e), suggesting that cardiomyocytes in this context are also not the primary protective targets of SR9009 treatment. Third, we investigated the cardiac fibroblasts as cellular targets by stimulating the cells with LPS in vitro, and then assaying formation and activity of the inflammasome. We show that whereas LPS induces expression of NLRP3 and IL-1β in cardiac fibroblasts, co-treatment with SR9009 protects against activation of the inflammasome (Fig. 4f). Along with this reduced inflammasome activity, there is also less mature IL-1β secreted from the cells (Fig. 4g). Thus collectively these data suggest that cardiac fibroblasts are a cellular target that contributes to the protective benefits of SR9009 in the heart.

### Timing of treatment (chronotherapy)

**Fig. 5:**
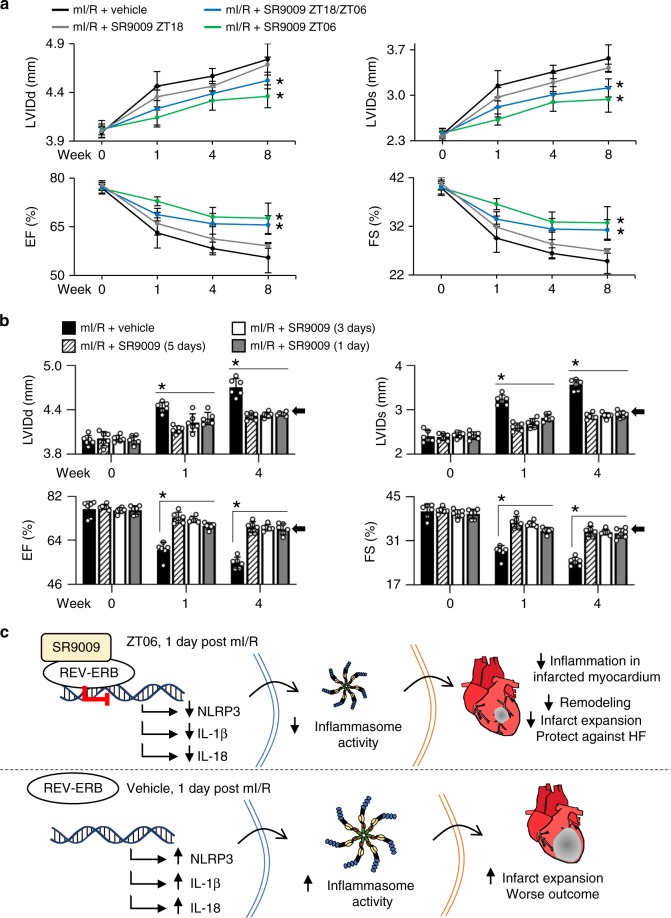
Treatment with SR9009 is time-of-day dependent, and treatment for just 1 day leads to improved outcomes post-mI/R. **a** Mice subjected to mI/R during their sleep time (ZT01-04) or wake time (ZT13-16) were chronotherapeutically treated with SR9009 at time points corresponding to the peak (ZT06) or trough (ZT18) of cardiac REV-ERB expression (see Supplementary Fig. 4). First or subsequent treatments at sleep time (ZT06) were most effective; see Supplementary Table 6 for all pathophysiology values. **b** Mice were subjected to mI/R (ZT01-04) and treated with SR9009 (ZT06) for 1, 3, or 5 days. Just 1 day of SR9009 treatment was sufficient to benefit outcomes versus vehicle-treated control; see Table 2 for pathophysiology data. **c** Proposed mechanism for the beneficial effects of targeting REV-ERB post-mI/R. SR9009 enhances REV-ERB repressor activity leading to reduced NLRP3 inflammasome priming and activation in the infarcted myocardium post reperfusion, leading to more efficient repair processes and protecting against HF

*Rev-Erbα* and *Rev-Erbβ* peak in the middle of murine sleep time (Supplementary Fig. 4) and thus we postulated that SR9009 treatment would be most effective around the time-of-day when its target is highest. To test this, mice were divided into four groups: (i) mI/R at sleep time, followed by SR9009 at reperfusion (ZT06, 5 days at ZT06); (ii) mI/R at wake time, followed by SR9009 at reperfusion (ZT18, then 5 days at ZT18); (iii) mI/R at wake time, followed by SR9009 at reperfusion (ZT18) and the following 5 days at ZT06; and (iv) mI/R at wake time, vehicle control. To assess the benefits of SR9009 on preventing progression to HF, mice were followed by pathophysiologic assessments for 8 weeks post-mI/R (Fig. 5a; Supplementary Table 6). We found that when mI/R occurred at sleep time, treatment at sleep time had the greatest benefit on healing, consistent with our notion that treatment corresponding to the peak time of Rev-Erb expression is most effective. When mI/R occurred at wake time, an initial treatment at the time of reperfusion followed by subsequent treatment at sleep time was similarly beneficial. This is important because humans may experience an MI at any time of day, and thus the first treatment can be given with reperfusion, and subsequently corresponding to the time of peak Rev-Erb expression, to maximize therapeutic benefits.

### SR9009 treatment for just 1 day protects against heart failure

Finally, we investigated the shortest treatment window needed to benefit long-term outcome. Since giving the drug at the time of reperfusion, and then just one subsequent day of SR9009 treatment post-mI/R attenuates the inflammasome (Fig. 3a–f), we postulated that only 1 day of treatment was needed to promote efficient healing long-term. To assess the minimum treatment regimen, mice were randomized to receive SR9009 at ZT06 at the time of reperfusion, followed by an additional treatment at ZT06 for 1 day, 3 days, or 5 days post-mI/R, and long-term outcomes were measured by pathophysiology. We found that just 1 day of SR9009 treatment significantly (*p* < 0.001) benefitted cardiac structure and function and prevented progression to HF (Fig. 5b, Table 2). Moreover, the benefits observed with just 1 day of SR9009 treatment were similar to those observed with 3 or 5 days of treatment, consistent with the notion that the benefits conferred occur within the first day after mI/R (black arrows, Fig. 5b). These findings are very promising because they suggest that only a very short treatment window of 1 day will reduce the adverse inflammasome, and create a better foundation to reduce infarct expansion, remodeling, and protect against heart failure (Fig. 5c).

**Table 2:** One day of SR9009 treatment benefits long-term cardiac structure and function post-mI/R

	mI/R + SR9009 (1 day)	mI/R + SR9009 (3 days)	mI/R + SR9009 (5 days)	mI/R + vehicle
*n*	6	6	6	6
*Echocardiography baseline*				
LVIDd (mm)	3.99 ± 0.02	4.01 ± 0.02	4.01 ± 0.04	3.99 ± 0.02
LVIDs (mm)	2.42 ± 0.03	2.43 ± 0.03	2.38 ± 0.03	2.40 ± 0.04
EF (%)	76.30 ± 0.72	76.06 ± 0.46	77.58 ± 0.43	77.12 ± 0.90
FS (%)	39.44 ± 0.61	39.45 ± 0.53	40.59 ± 0.39	40.32 ± 0.72
HR (bpm)	460 ± 13	483 ± 8	476 ± 3	467 ± 4
BW (g)	22.73 ± 0.77	23.38 ± 0.80	21.90 ± 0.35	23.42 ± 0.97
*1 week post-mI/R*				
LVIDd (mm)	4.28 ± 0.03	4.23 ± 0.03	4.13 ± 0.02	4.43 ± 0.02**
LVIDs (mm)	2.82 ± 0.02	2.70 ± 0.02	2.61 ± 0.03	3.22 ± 0.04**
EF (%)	69.80 ± 0.40	72.40 ± 0.37	73.18 ± 0.71	59.91 ± 1.03**
FS (%)	34.20 ± 0.30	36.27 ± 0.29	36.80 ± 0.59	27.46 ± 0.63**
HR (bpm)	463 ± 6	480 ± 6	464 ± 6	456 ± 6
*4 weeks post-mI/R*				
LVIDd (mm)	4.34 ± 0.01	4.33 ± 0.01	4.31 ± 0.01	4.71 ± 0.04**
LVIDs (mm)	2.90 ± 0.02	2.87 ± 0.02	2.85 ± 0.02	3.56 ± 0.04**
EF (%)	68.51 ± 0.66	69.09 ± 0.37	69.25 ± 0.71	54.73 ± 0.73**
FS (%)	33.34 ± 0.46	33.71 ± 0.26	33.82 ± 0.52	24.32 ± 0.42**
HR (bpm)	448 ± 6	464 ± 8	470 ± 3	471 ± 4
*Morphometry*				
HW (mg)	125.67 ± 3.62	124.50 ± 2.79	123.33 ± 1.41	135.67 ± 1.88**
HW:BW (mg g^−1^)	4.50 ± 0.09	4.52 ± 0.06	4.36 ± 0.11	4.73 ± 0.04*
HW:TL (mg mm^−1^)	6.19 ± 0.15	6.15 ± 0.13	6.11 ± 0.07	6.68 ± 0.11**
BW (g)	27.90 ± 0.59	27.52 ± 0.52	28.35 ± 0.82	28.69 ± 0.44

*LVIDd* left ventricle internal dimensions at diastole, *LVIDs* LV internal dimensions at systole, *% EF* % ejection fraction, *% FS* % fractional shortening, *HR* heart rate, *HW* heart weight, *BW* body weight, *TL* tibia length

*p  <  0.05, **p <  0.01, mI/R + vehicle vs. all groups by ANOVA. Values are mean ±  SEM

Collectively, we show using both pharmacologic and genetic approaches that short-term targeting of the circadian mechanism in vivo and post-mI/R benefits outcome. As illustrated in Fig. 5c, SR9009 targets *Rev-Erb* and activates repressor activity leading to decreased priming of genes under its transcriptional control. This includes the Nlrp3 inflammasome, leading to reduced adverse innate and adaptive post-reperfusion inflammatory responses, thereby protecting against infarct expansion and left ventricular remodeling, and thus benefiting heart structure and function and protecting against heart failure.

## Discussion

We found, in a murine model of mI/R using pharmacological and genetic approaches, that as little as 1 day of targeting of the circadian mechanism in vivo and post-mI/R reduces infarct expansion and prevents heart failure. As illustrated in Fig. 5c, our data show that SR9009 treatment at ZT06 for just 1 day post-mI/R enhanced REV-ERB repressor activity and led to downregulation of cardiac NLRP3, IL-1β, and IL-18. These are critical constituents of the NLRP3 inflammasome; SR9009 treatment reduced inflammasome activity. Downregulation of the inflammasome subsequently limited recruitment of inflammatory cells and mediators in infarcted myocardium, and laid a better foundation for healing, resulting in reduced infarct expansion and protection against HF. In contrast, the untreated mI/R mice developed infarct expansion, adverse LV remodeling, and clinically worse outcomes.

To our knowledge, this is the first study using pharmacological targeting of the circadian mechanism to reduce inflammasome activity and inflammatory cells recruited to infarcted myocardium after reperfusion, leading to reduced cardiac remodeling and infarct expansion, and protecting against heart failure in vivo. Previously, we and others showed that Rev-Erb agonism with SR9009 reduces cardiac hypertrophy in pressure overload-induced cardiac hypertrophy in mice^14,15^. Also pre-treatment of mice with SR9009 before acute myocardial infarction provides protective benefits^16^, although long-term prophylactic treatment in real life may not be feasible. Montaigne et al. reported a time-of-day-effect on outcomes for patients undergoing aortic valve replacement; their study linked Rev-Erb antagonism with upregulation of genes involved in cardiac remodeling ex vivo^17^. Here, we use the clinically relevant myocardial ischemia reperfusion model (mI/R), which simulates the benefits of pharmacological targeting of the circadian mechanism on the evolution of ventricular remodeling after heart attacks in humans. We show that Rev-Erb agonism reduces formation of the adverse NLRP3 inflammasome in vivo. Our approach is especially promising because treatment is given only after myocardial infarction occurs, and for only 1 day, and can be given alongside conventional therapies such as reperfusion to improve healing. Because treatment limits the early adverse reperfusion injury, then the infarct region of the LV is capable of intrinsically healing, and subsequent LV remodeling and progression to heart failure (HF) is prevented, which has important implications for patients that could lead to longer and healthier lives.

Mechanistically, our results show that the benefits of pharmacologically targeting the circadian mechanism coincide with an observed decrease in the NLRP3 inflammasome. The inflammasome is a natural damage sensor that is activated in response to noninfectious stimuli such as cardiomyocyte debris following infarction. It triggers the immune system to clear the debris; however, in doing so it is well documented to exacerbate myocardial damage via local immune cell responders and released cytokines and other mediators^18,19^. As has been elegantly reviewed by Toldo and Abbate^20^, recent experimental studies have focussed on the NLRP3 inflammasome as an important new therapeutic target to reduce reperfusion injury, infarct size, and prevent the development of HF following myocardial infarction. However, no selective NLRP3 inhibitors are clinically available for use in patients at present, indeed studies identifying selective NLRP3 inhibitors are eagerly anticipated^20^. Our findings here reveal very promising results that SR9009 treatment for just 1 day decreases formation of the cardiac NLRP3 inflammasome, leading to reduced infarct expansion, and prevention of HF in mice.

There are several lines of evidence supporting a direct link between pharmacologically targeting the circadian factor REV-ERB and downregulation of the NLRP3 inflammasome. Previous studies by others showed that REV-ERB is a transcription factor that negatively regulates NLRP3 in macrophages^21,22^, as well as transcription of the proinflammatory cytokine IL-1β that is subsequently processed by NLRP3 inflammasome activity^21^. Treatment with the Rev-Erb agonist SR9009 decreases NLRP3 inflammasome in macrophages^21,22^. Conversely, it has been shown that loss of REV-ERB leads to increased NLRP3, IL-1β, and IL-18 in macrophages using REV-ERB knockout (*Nr1d1*^*−/−*^) mice^21,22^. Moreover, here, we show that REV-ERB also appears to regulate the NLRP3 inflammasome in the infarcted heart. First, treatment with SR9009 reduces the inflammasome and improves HF outcomes in WT mice as compared with vehicle-treated controls. Second, we found that treatment with the Rev-Erb agonist SR9009 reduces Rev-Erb mRNA and protein in the normal heart, and in the infarcted heart, and reduces NLRP3 mRNA and its constituent cytokine mRNA’s in the infarcted heart. Third, we found no protective benefits of SR9009 on HF outcomes in *Nr1d1*^*−/−*^ mice lacking the REV-ERB target, consistent with the notion that a functional REV-ERB target is necessary for SR9009 efficacy. Fourth, SR9009 treatment only attenuated inflammasome expression in WT mice with a functional REV-ERB target, but not in the *Nr1d1*^*−/−*^ mice lacking the REV-ERB target. Fifth, loss of REV-ERB led to increased expression of Nlrp3, Il-1β, and Il-18 in *Nr1d1*^*−/−*^ cardiac fibroblasts in vitro, consistent with a loss of repressor activity. Sixth, SR9009 more effectively reduced inflammasome expression in LPS-treated cardiac fibroblasts from WTs as compared with *Nr1d1*^*−/−*^ cells lacking the REV-ERB target. Finally, targeting REV-ERB is effective by day 1 and at sleep time, corresponding to when REV-ERB is most abundant. Thus collectively, these data support the notion that REV-ERB is a critical regulator of the NLRP3 inflammasome, and thus a promising target for reducing inflammasome-mediated reperfusion injury in the heart in the first few days post-mI/R to improve outcomes.

It is important to note that in this study we are not preventing inflammation, only turning down the immune responses. A balance is important because immune cells exhibit divergent functions, and some responses are important for infarct repair^6,7,11,23–26^. For example, the early responding neutrophils deposit MPO, a potent enzyme to break down cellular debris, but this also becomes involved in adverse remodeling after myocardial injury^27–29^. Indeed, chronically elevated plasma MPO levels found in human patients following ST-elevation myocardial infarction (STEMI) are a predictor of poor outcome^30^. Here, we show that reducing the NLRP3 inflammasome leads to less reperfusion injury: a decreased cascade of the neutrophil, macrophage, and T cell recruitment to the infarct region. The LV infarct can better heal, resulting in less adverse remodeling, and protection against HF.

REV-ERB is a strong regulator of the inflammasome pathway, and thus it presents as an ideal target for reducing reperfusion injury responses post-mI/R. SR9009 exhibits specificity for REV-ERB, as the agonist has been pharmacologically tested against 48 nuclear receptors and shown to be selective for REV-ERB in these studies^13^. Moreover, it has been tested in the NIH psychoactive drug specificity panel (PDSP, https://pdsp.unc.edu) against an array of GPCRs, transporters, and ion channels, with no significant cross-reactivity^13^. This specificity is consistent with our genetic data for loss and gain of function in the circadian mutant and knockout mice. Interestingly, REV-ERB also binds naturally occurring heme to modulate its activity^31,32^, Notably, reperfusion hemorrhage with elevated heme levels is strongly associated with worse outcomes post-mI/R^33,34^; thus it is tempting to speculate that SR9009 may also work indirectly through displacement of heme to provide additional benefits to negate reperfusion injury. This notion would be consistent with a recent report^35^, suggesting that the benefits of SR9009 may be attributed to strategies in addition to direct modulation of REV-ERB activity. It would be interesting to look at the relative effects on heme displacement post-mI/R using REV-ERBα and/or REV-ERBβ mice in future studies. In summary, these data identify REV-ERB as a pharmacological target to downregulate the NLRP3 inflammasome, and possibly additional adverse immune responses, for mitigating MI reperfusion injury and preventing HF.

MI is a well-recognized illness that triggers pathophysiologic cascades leading to HF. There is an urgent public health need to prevent HF; it is a leading cause of hospitalization, an enormous economic burden, and there is no known cure^36–39^. Infarct size is a powerful predictor of death^40^. Infarct expansion can be reduced by myocardial post-ischemia reperfusion, which is the timely restoration of blood flow to ischemic myocardium, for example through percutaneous coronary intervention or angioplasty^41,42^. However, even if reperfusion is successful, this triggers reperfusion injury; an adverse inflammatory response and many patients continue to develop adverse remodeling leading to HF^5,42^. Previously, we and others showed that the circadian mechanism plays a critical role in cardiac inflammation post-MI^8,10,43,44^. This study now reveals that circadian pharmacology can target the inflammasome to reduce reperfusion injury, presenting the possibility that we can intrinsically heal an MI. Notably, ischemia and reperfusion injury also contribute to a wide range of cardiovascular pathologies (e.g., following organ transplantation, hypoxia, valve replacement, post-ischemic arrhythmias)^3,29^, suggesting potential for additional important therapeutic applications of targeting REV-ERB even beyond ischemic heart disease and infarct repair. With the aging of our population, and the emerging epidemic of cardiovascular disease, circadian medicine strategies hold promise for reducing cardiovascular morbidity and mortality, leading to longer healthier lives.

Collectively, our studies shed light on a critical role for REV-ERB in cardiac repair, and illustrate the importance of targeting the circadian mechanism to reduce reperfusion injury post-MI and prevent the development of HF. Our pharmacological approach reduces adverse inflammasome activation, thereby limiting cytokine and immune cell recruitment to the infarcted myocardium, especially during the period of time when the infarct is highly active biologically. This reduces stress on the vulnerable infarct, limiting the profound and deleterious changes in LV structure, and benefits function after mI/R. These studies have been done in mice, which bear some limitations, but at a minimum the promising experimental results focus expectations toward pharmacology that can benefit patients with heart disease. We hope our experimental study will stimulate the initiation of translational research targeting REV-ERB, and indeed explore an emerging class of drugs that target the circadian mechanism, to benefit treatment of patients clinically.

## Methods

### Experimental design

All animal work was conducted under the guidelines of the Canadian Council on Animal Care. Briefly, 8-week-old C57BL/6 mice (Charles River) were housed in a 12-h light (L) and 12-h dark (D) cycle (12:12 LD) for 2 weeks prior to surgery. Mice were anaesthetized with isoflurane, intubated and ventilated, and subjected to left anterior descending coronary artery ligation for 45 min, followed by reperfusion (mI/R model), and then maintained for 8 weeks (heart failure (HF) model). Sham animals underwent the same surgical procedures, but without coronary artery ligation. All surgeries were performed between ZT01 and ZT04, unless otherwise indicated, and all samples were collected for molecular analyses at ZT07 unless otherwise noted. Following recovery from mI/R, the mice were randomized to one of two groups. Either (i) they were given SR9009 (100 mg per kg in 15% cremophor, once daily at ZT06), or (ii) were given vehicle only (15% cremophor). Dosage was based on murine studies of in vivo efficacy^13,14,45^. Following treatment (for 1 to 5 days as described in the studies), all groups were maintained without any further drug treatments for up to 8 weeks post-mI/R. To show that all infarcts started the same, hearts were collected within the first day, and Evans Blue and 2,3,5-triphenyltetrazolium chloride (TTC) staining was performed. Mice were then followed for pathophysiologic assessments to determine the benefits of SR9009 targeting of *Rev-Erb* on outcomes including by echocardiography (at baseline, 1, 4, and 8 weeks post-mI/R), morphometrics, in vivo pressure–volume hemodynamics (8 weeks post-mI/R) and pathologic measures of infarct volume and infarct expansion (8 weeks post-mI/R). Hearts from a separate set of mice were collected for examining the effects of SR9009 on *Rev-Erb* cardiac gene and protein expression. A third set of hearts were collected for examining how targeting *Rev-Erb* modulated cytokine and inflammasome responses at 1, 2, 3, 5, 7, and 14 days post-mI/R by real-time polymerase chain reaction (RT-PCR). A fourth set of mice was used to quantify how targeting *Rev-Erb* affected cardiac immune cell infiltration post-mI/R to benefit outcome (cardiac myeloperoxidase marker of myeloid cells; total leukocytes, macrophages, and T cell activation and infiltration). A fifth set were REV-ERB (*Nr1d1*^*−/−*^) knockout mice on a C57BL/6 background^46^, that were maintained in our breeding colony. They were used to investigate the effects of SR9009 treatment post-mI/R in the absence of REV-ERBα activity. A sixth set were *Clock*^Δ19/Δ19^ mice^47^ (these are homozygous for the CLOCK point mutation) bred on a C57Bl/6 background, our breeding colony is maintained in the Central Animal Facilities at the University of Guelph^10,14^. These and wild-type (WT) controls were used to investigate the link between SR9009 treatment and REV-ERB activity for the benefits of targeting the circadian mechanism to improve outcomes after mI/R. These animals were divided into sets for use for determination of gene expression, infarct size after mI/R, pathophysiology and outcome, and quantification of leukocyte infiltration to the infarcted myocardium, as described above. A seventh set of mice were used to investigate how time-of-day and length of treatment influence cardiac inflammation and remodeling and improved outcomes in HF. For the time-of-day chronotherapy studies, a subset of mice were killed every 4 h for 24 h, and hearts collected to determine the diurnal profile and peak and trough timing of REV-ERB in the heart. All endpoints assessed and *n*-values and statistics are provided in detail in the Figure legends and the Supplementary information.

### Animals

Male C57Bl/6 mice (Charles River, Quebec, Canada), and *Clock*^*Δ19/Δ19*^ mice^47,48^ (homozygous for the CLOCK point mutation, C57Bl/6 background) and *Nr1d1*^*−/−*^ mice^46^ (null mutation of *Rev-Erb*, C57Bl/6 background) were housed in a 12 h light (L):12 h dark (D) cycle with lights on at 9:00 am (Zeitgeber time 0, ZT0) and lights off at 9:00 pm (ZT12). All animals were housed at the Central Animal Facility, University of Guelph. Standard rodent chow and water were provided ad libitum throughout the study. All studies were approved by the University of Guelph Institutional Animal Care and Use Committee, and the Canadian Council on Animal Care guidelines. *Clock*^*Δ19/Δ19*^ and *Nr1d1*^*−/−*^ mice were genotyped by allele-specific PCR (see Supplementary Table 7 for primers). Genotyping, and phenotyping of circadian locomotor activity using running wheel actigraphy, was previously described^14,48,49^. Individually housed mice were entrained to a diurnal 12:12 L:D cycle for 2 weeks, followed by transfer to constant darkness (circadian cycle, DD). The data were analyzed using ClockLab (ActiMetrics).

### Myocardial ischemia/reperfusion

Eight-week-old mice (22-25 g) underwent surgical ligation of the left anterior descending (LAD) coronary artery followed by reperfusion. All surgeries were performed between ZT01-04, unless otherwise described for the time-of-day experiments; for these, surgeries were either done during the murine sleep time (ZT01-04) or murine wake time (ZT13-16) using our established protocols^43^. Mice were anaesthetized with isoflurane, intubated, and ventilated (Harvard Apparatus model 687; St Laurent, Quebec, Canada). Prior to incision, animals were administered a local subcutaneous injection of a 50:50 mix of bupivacaine (1.5 mg per kg) and lidocaine (3 mg per kg). A left-sided incision was made in the third intercostal space to view the heart, and the pericardium was gently dissected to expose the LAD. A silk 7–0 suture (Ethicon) was passed underneath the LAD at 1 mm below the left auricle, and a 5 mm piece of PE-10 tubing was placed over the LAD. The suture was tied around the tubing and LAD to induce ischemia for a 45- min period. The chest was temporarily closed, and a sterile cloth placed over the incision site. After 45 min, the site was re-exposed and the tubing and suture were removed to allow for LAD reperfusion. The chest and skin were then closed using a silk 6–0 suture (Ethicon). Sham-operated animals underwent the same procedure but the LAD was not ligated. Mice were administered buprenorphine (0.1 mg per kg) analgesia upon awakening, and again at 8  and 24 h postoperatively. Mice were randomized to either drug treatment or vehicle control, as described below.

### Evans blue and TTC staining

Following reperfusion, mice were anaesthetized, intubated, and ventilated as described above. For infarct size quantification, the suture was left in the heart during mI/R surgery to allow the ligature to be re-tied at the same site for infarct size assessment. A 1% Evans Blue solution was infused through the inferior vena cava to visualize the ischemic myocardium. The heart was removed, rinsed in 0.9% saline, and sectioned into 1 -mm slices using a heart matrix (Zivic Instruments). Slices were incubated in 1% TTC solution for 10 min, transferred to 10% neutral buffered formalin for 90 min, and photographed. Infarct area and area at risk (AAR) as a percentage of left ventricle (LV) were determined as described previously^10,43^. Briefly, infarct area and area at risk (AAR) as a percentage of left ventricle were determined using Adobe Photoshop CS4. Samples were normalized to each slice weight with the formula: weight (total AAR) = [weight (slice 1) × % AAR (slice 1)] + [weight (slice 2) × % AAR (slice 2)] + [weight (slice *n*) × % AAR (slice *n*)]. Infarct area was calculated in a similar manner. Absolute infarct size was calculated as a ratio of weight (total infarct area)/weight (total AAR).

### SR9009 treatment

The REV-ERB agonist SR9009 treatment was given in a blinded manner, intraperitoneally (i.p.) at a dose of 100 mg per kg in 15% cremophor, versus vehicle (15% cremophor), to mice after mI/R. The first treatment was given at ZT06 following reperfusion, then once daily for 5 days, and then all animals were maintained without any treatments for up to 8 weeks after mI/R. Dosage was based on murine studies of in vivo efficacy^13,14,45,50^. In a separate study investigating only normal healthy hearts, mice were injected i.p. at ZT06 once per day for 2 weeks. A separate cohort of mice used to investigate the time-of-day effects of SR9009 treatment post-mI/R were given the drug at ZT18 at reperfusion and then for all 5 days, or ZT18 post-reperfusion followed by ZT06 once daily for 5 days. Another experiment investigated whether treatment was effective if given at ZT06 for 1, 3, or 5 days.

### Echocardiography

To assess cardiac function and morphometry, animals were assessed at baseline, 1, 4, and 8 weeks post-mI/R, in a blinded manner under light anesthesia (1.5% isoflurane) using a GE Vivid 7 Dimension ultrasound machine (GE Medical Systems) with a i13L14 MHz linear-array transducer. Measurements were taken at the mid-papillary level and used to determine left ventricular internal dimension at end-diastole (LVIDd), left ventricular internal dimension in systole (LVIDs), % ejection fraction (% EF), % fractional shortening (% FS), and heart rate (HR). A minimum of five images per mouse heart were analyzed.

### Pressure–volume hemodynamics in vivo

Hemodynamics measurements were collected at 8 weeks post surgery. Animals were anesthetised using 4% isoflurane, intubated, and hemodynamic measurements were taken as previously described^10,14,51–53^. Body temperature was continuously monitored and maintained. The carotid artery was isolated, a small incision was made, and a 1.2Fr pressure volume catheter (Transonic) was advanced into the LV via the ascending aorta. Physiologic LV and aortic pressure measurements were recorded on an ADInstrument PowerLab. Left ventricular end systolic pressure (LVESP), diastolic pressure (LVEDP), systolic volume (LVESV), diastolic volume (LVEDV), stroke volume (SV), cardiac output (CO), maximum and minimum first derivatives of LV pressure (dP/d*t*_max_; dP/d*t*_min_), systolic/diastolic blood pressures (SBP/DBP) were recorded. Mean arterial blood pressure (MAP) was calculated as DBP + (SBP-DBP)/3. Continuously recorded pressures were analyzed with Lab Chart 7 (Colorado Creeks, USA).

### Histology

At 8 weeks post-mI/R, mice were euthanized with isoflurane and cervical dislocation. Body weight (BW), heart weight (HW), and tibia length (TL) were collected for each animal. Hearts were perfused with 1 M KCl, fixed in 10% neutral buffered formalin for 24 h, and paraffin embedded. Hearts were sectioned (5 μm) from apex to base, collecting ten sections every 300 μm. Sections were stained with Masson’s trichrome for infarct quantification. Infarct volume was calculated as a percentage of LV volume. Relative infarct expansion was determined by dividing the sum of the endocardial and epicardial circumference occupied by the infarct by the sum of the total LV epicardial and endocardial circumferences. *N* = 5 hearts/group, with images taken by using Q-Capture (QImaging; Surrey, BC) and analyzed in Image J 1.46 (NIH).

### RNA isolation and RT-PCR

Total RNA was isolated from hearts using TRIzol (Invitrogen) according to the manufacturer’s instructions, and quality assessed by Nanodrop (Thermo Scientific). RT-PCR was performed on a VIIA7 real-time PCR system (Life Technologies) using the RNA-to-Ct one step PCR kit (ThermoFisher Scientific) under the following protocol: reverse transcription, 48 °C for 30 min, 95 °C for 10 min for one cycle, followed by amplification at 95 °C for 15 s, 60 °C for 1 min for 40 cycles, followed by hold at room temperature. The primers for *nuclear receptor subfamily 1, group D1* (*Rev-Erbα*), *nuclear receptor subfamily 1, group D2* (*Rev-Erbβ*), *circadian locomotor output cycles protein kaput* (*Clock*), *interleukin 6* (*Il-6*), *chemokine (C-C motif) ligand 2 (Ccl2)/macrophage chemotactic protein 1 (Mcp1*), *Ccl7*, *NLR Family Pyrin Domain 3 (Nlrp3), Il-1β, Il-18*, and *histone* are listed in Supplementary Table 7. RT-PCR was normalized to *histone* using the ΔΔCT method, as described previously^10,43,54,55^.

### Myeloperoxidase (MPO) assay

A separate set of hearts were collected at 1, 2, and 3 days post-mI/R for assessment of myeloid cell infiltration of the infarcted myocardium, by MPO assay, as previously described^10^. Briefly, animals were killed by isoflurane and cervical dislocation, and hearts were rinsed in phosphate buffered saline, weighed, and stored at −80 °C until use. MPO was isolated using the EnzChek Myeloperoxidase Activity Assay Kit (Life Technologies; Molecular Probes). Tissue was homogenized in 1 mL of 50 mM potassium phosphate buffer (pH 6.0) with 0.5% (w/v) hexadecyltrimethyl ammonium bromide. The homogenate was sonicated and freeze-thawed three times, then centrifuged (20,000 rpm, 15 min) using a Beckman Ultracentrifuge (LE-80K). The supernatant containing MPO was collected and quantified. Values were normalized to tissue weight. *n* = 5 hearts/group, and all samples were run in duplicate.

### Cell tracking

Cell tracking experiments were performed as previously described^23^. Briefly, 0.5 μm of fluoresbrite microspheres (Polysciences) were opsonized with 50% mouse serum:PBS, and injected into the infarct border zone in the LV anterior free wall during mI/R or sham surgery. The myocardium, peripheral lymph nodes (mediastinal, axillary, cervical, and inguinal), spleen, and bone marrow were collected 24 h later and analyzed by flow cytometry.

### Immune cell quantification by flow cytometry

A separate set of hearts was collected to quantify immune cell recruitment to the infarcted myocardium post-mI/R. Briefly, animals were killed as described above. Hearts were exposed, perfused via cardiac puncture with 100 units heparin in 5 mL saline, and then removed and rinsed in saline. Epicardial fat, atria, and the free wall of the right ventricle were removed, and the left ventricle was minced into 1–2 mm pieces with a razor blade. Hearts were digested for 60 min at 37 °C with collagenase type II (1 mg per mL; Worthington Biochemical Corporation) and DNase I (60 U per mL; Roche Diagnostics Corporation) in 10 mL RPMI 1640 media (Life Technologies). Released cells were separated using a 70-μm cell strainer (Fisher Scientific). Cell count and viability (> 90%) were determined using the trypan blue exclusion method. Aliquots were diluted to 1 × 10^6^ cells in 50 μl staining buffer (PBS, 2% BSA) and incubated with anti-CD16/32 (clone 93, eBioscience) for 5 min at 4 °C to block Fcγ receptors. After blocking, cell suspensions were incubated with fluorochrome-conjugated antibodies in 50 μl of staining buffer in the dark for 30 min at 4 °C. To examine immunocyte recruitment cells were stained for total leukocytes with anti-CD45-APC (clone 30-F11, eBioscience), or for macrophages with anti-CD11b-PE (clone M1/70, Biolegend) and anti-F4/80-FITC (clone BM8, Biolegend) and anti-CD206-AlexaFluor647 (clone C068C2, Biolegend; M1 = 206-, M2 = 206+), or for T cells with anti-CD3e-FITC (clone 145-2C11, Biolegend) and anti-CD4-PE (clone GK1.5, Biolegend), or anti-Ki67-APC (clone 16A8, Biolegend). Samples were analyzed on an Accuri C6 Flow Cytometer (BD Biosciences) using BD Accuri C6 Software (BD Biosciences) and FlowJo software (Tree Star). *N* = 5 hearts/group.

### Irradiation and bone marrow transplantation (BMT)

Experiments were performed using established protocols^56^. Briefly, the recipient mice (WT CD45.1^+^, 8 weeks old) were lethally irradiated with a total dose of 9 Gy (1 Gy per min dose rate; Clinac iX linear accelerator, Varian Medical Systems) and the following day injected with 6–10 × 10^6^ bone marrow cells from donor mice (WT CD45.2^+^, or *Nr1d1*^*−/−*^ CD45.2^+^) through the tail vein. The donor cells had been freshly harvested from mice killed by isoflurane and cervical dislocation, by flushing femurs and tibiae with PBS. Irradiated and reconstituted mice were housed in a strict barrier environment in autoclaved microisolator cages. Immune cell reconstitution of recipient mice was validated as > 96% by flow cytometry at 8 weeks post-BMT using anti-mouse CD45.1 (APC, clone A20, Biolegend) and anti-mouse CD45.2 (PE, clone 104; Biolegend). Using this protocol, we produced two types of BMT mice: WT to WT (BMT^WT→WT^) mice and Nr1d1^*−/−*^ to WT (BMT^Nr1d1*−/−*→WT^) mice.

### Cardiac myocyte and fibroblast cultures

Neonatal mouse cardiomyocytes and fibroblasts were cultured using standard techniques^14^. Cells were plated at a density of 500 cells per mm^2^ in the Dulbecco’s Modified Eagles Medium/F12 (DMEM; ThermoFisher Scientific) with 10% fetal bovine serum (FBS; ThermoFisher Scientific), 0.1% bromodeoxyuridine (BrdU; Sigma). After 24 h, the cardiomyocyte cultures were changed to serum-free DMEM containing 1% transferrin (Sigma), insulin (Sigma) and BrdU, and SR9009 (dissolved in dimethyl sulfoxide, DMSO; Sigma) at 0 µM, 2.5 µM, 5 µM, or 10 µM. Plates were incubated under normal (5% CO_2_/95% Air) or hypoxic (5% CO_2_/95% N_2_) conditions at 37 °C for 4 h and then under normoxia conditions at 37 °C for 18 h. Cardiomyocyte cultures were assessed for cell viability by lactate dehydrogenase assay (Cytotoxicity Detection Kit Plus, Roche), as per the manufacturer’s directions. For the neonatal fibroblast cultures, cells were plated for 24 h and then changed to media containing 100 ng per mL LPS+/– SR9009, or vehicle controls for 24 h. Supernatant was collected for ELISAs, and cells were collected into CellLytic MT Cell Lysis Reagent (Sigma) for western blots. Adult mouse cardiac fibroblasts were cultured in 60 -mm dishes by the method of Wang et al.^57^ for 24 h, and then treated with LPS+/– SR9009 as described above, and cells were collected into 700 µl of TRIzol (Invitrogen) for RNA isolation and RT-PCR of *Nlrp3*, *Il-1β*, and *Il-18*.

### Western blots

Protein lysates were prepared using CelLytic MT Cell Lysis Reagent (Sigma) as described previously^14^, then subjected to SDS-PAGE, with the Precision Plus Protein Dual Color Standard marker (Bio-Rad) and transferred to PVDF membrane (Bio-Rad). Primary antibodies used were REV-ERBα (mouse monoclonal, clone 4F6; 1:1000; Abgent), or REV-ERBβ (mouse monoclonal, clone D-8; 1:1000; Santa Cruz Biotechnology), or NLRP3 (mouse monoclonal, clone Cryo-2; 1:1000; Adipogen), or IL-1β (rabbit polyclonal, GTX74034; 1:1000; GeneTex), and incubated overnight at 4 °C. β-actin (mouse monoclonal, clone C4; 1:10,000; Millipore) was used as a loading control. Membranes were washed with tris-buffered saline with 0.1% Tween-20 (TBS-T), and incubated for 1 h at room temperature with horseradish peroxidase-conjugated secondary antibodies (anti-mouse, 7076S, 1:5000; anti-rabbit, 7074S, 1:5000; Cell Signaling) in 3% skim milk in TBS-T. Proteins were visualized using Clarity Western ECL substrate (Bio-Rad), according to the manufacturer’s specifications. Densitometry quantification was performed using Image J 1.46 (NIH). All original blots are provided in Supplementary Fig. 5.

### ELISAs

Heart protein lysates or cardiac cell culture supernatants were assayed for IL-1β (BMS6002, Invitrogen) or IL-18 (BMS618/3, Invitrogen), according to the manufacturer’s instructions.

### Statistics and reproducibility

Values are expressed as mean ± SEM. Statistical comparisons were done using an unpaired two-sided *S*tudent’s *t* test or a one-way or two-way analysis of variance (ANOVA) followed by Tukey post hoc for multiple comparisons, as applicable. Values of *p* ≤ 0.05 are considered statistically significant. All endpoints assessed and *n*-values and statistics are provided in detail in the Figure legends. All graphs are plotted as standard deviation to show the spread and variability of the data, or standard error with individual data points for comparing the differences between means.

### Reporting summary

Further information on research design is available in the Nature Research Reporting Summary linked to this article.

## Supplementary information

Supplementary Information

Description of Additional Supplementary Files

Supplementary Data 1

Reporting Summary

## Data Availability

The data that support the findings of this study are available in the Supplementary Information, or from the corresponding author on reasonable request. The source data underlying the main figures are shown in Supplementary Data 1.
