# Supplementary material for: SR9009 administered for one day after myocardial ischemia-reperfusion prevents heart failure in mice by targeting the cardiac inflammasome
Source: Commun Biol. 2019 Oct 3;2:353. doi: 10.1038/s42003-019-0595-z (PMC6776554; doi:10.1038/s42003-019-0595-z)
Supplement: Supplementary file 1 — Supplementary Information [file 42003_2019_595_MOESM1_ESM.pdf]

Supplementary Figure 1

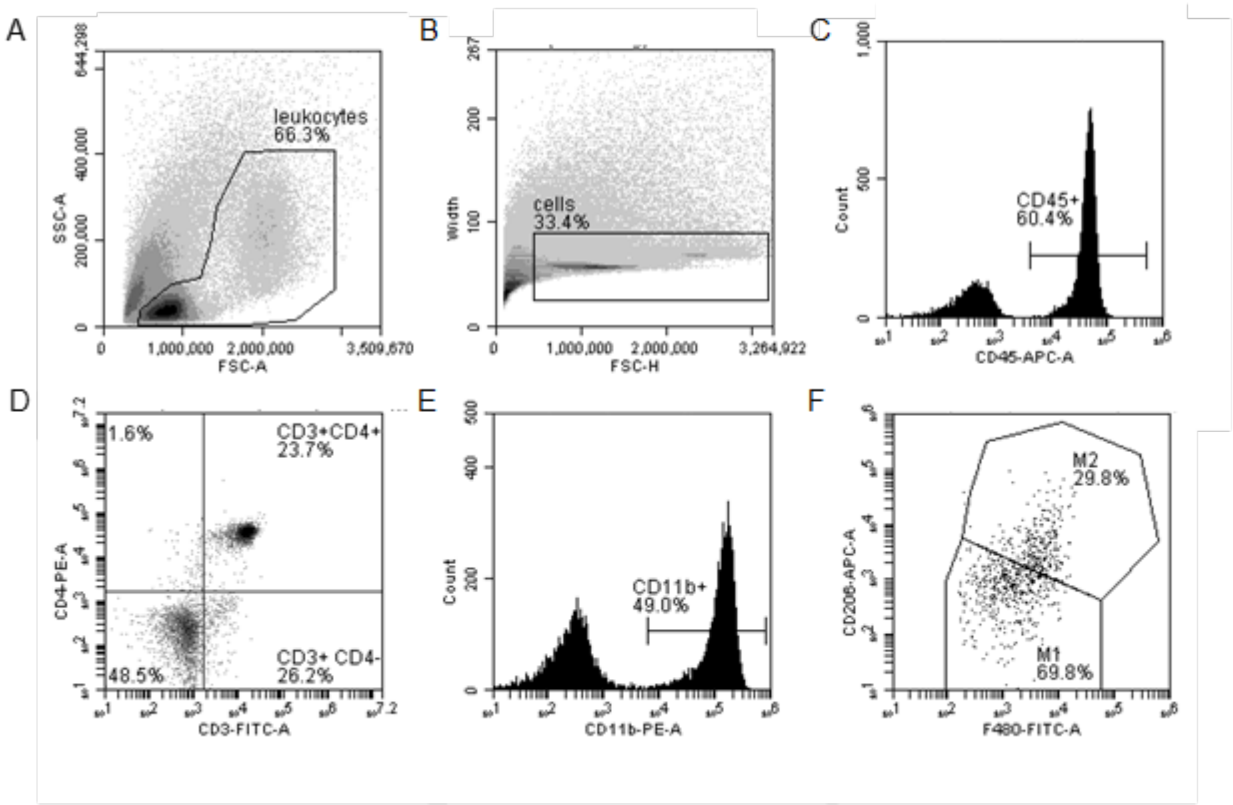

Supplementary Figure 1: Representative gating strategy for flow cytometry analyses. Cells from mouse heart, lymph nodes, spleen, blood, or bone marrow were analyzed for leukocyte surface antigens using flow cytometry techniques. (A) The gating strategy incorporated the selection of cells, and (B) removed the debris. (C) From this gating strategy, CD45+ leukocytes were selected, and transposed into a histogram. (D) The CD45+CD3+CD4+ subset was selected from that population. (E) From the gating strategy, CD11b+ macrophages were selected and transposed into a histogram. (F) The M1 (F4/80+CD206-) and M2 (F4/80+CD206+) subpopulations of CD11b+ macrophages were selected from that population.

Supplementary Figure 2

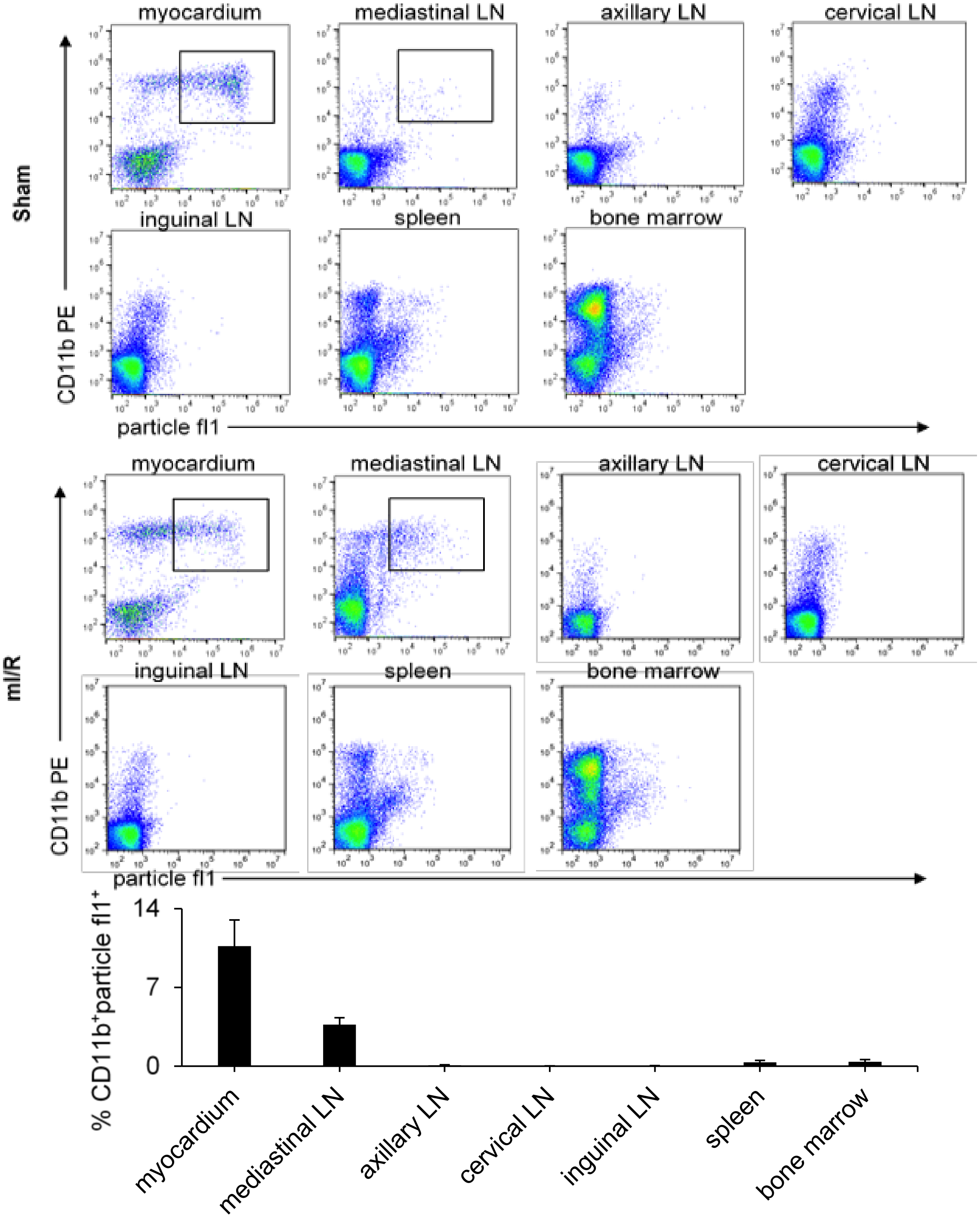

Supplementary Figure 2: Leukocytes from the heart drain to the mediastinal lymph node (LN) post-mI/R.

Fluorescent microparticles were injected into the anterior free wall of the LV during mI/R or sham surgery. After 24 hours, cell suspensions from heart tissue, LN, spleen, or bone marrow were analyzed by flow cytometry analyses. Sham mice (top), mI/R mice (middle), CD11b+ leukocyte trafficking from n ≥ 4 mice/group (bottom).

Supplementary Figure 3

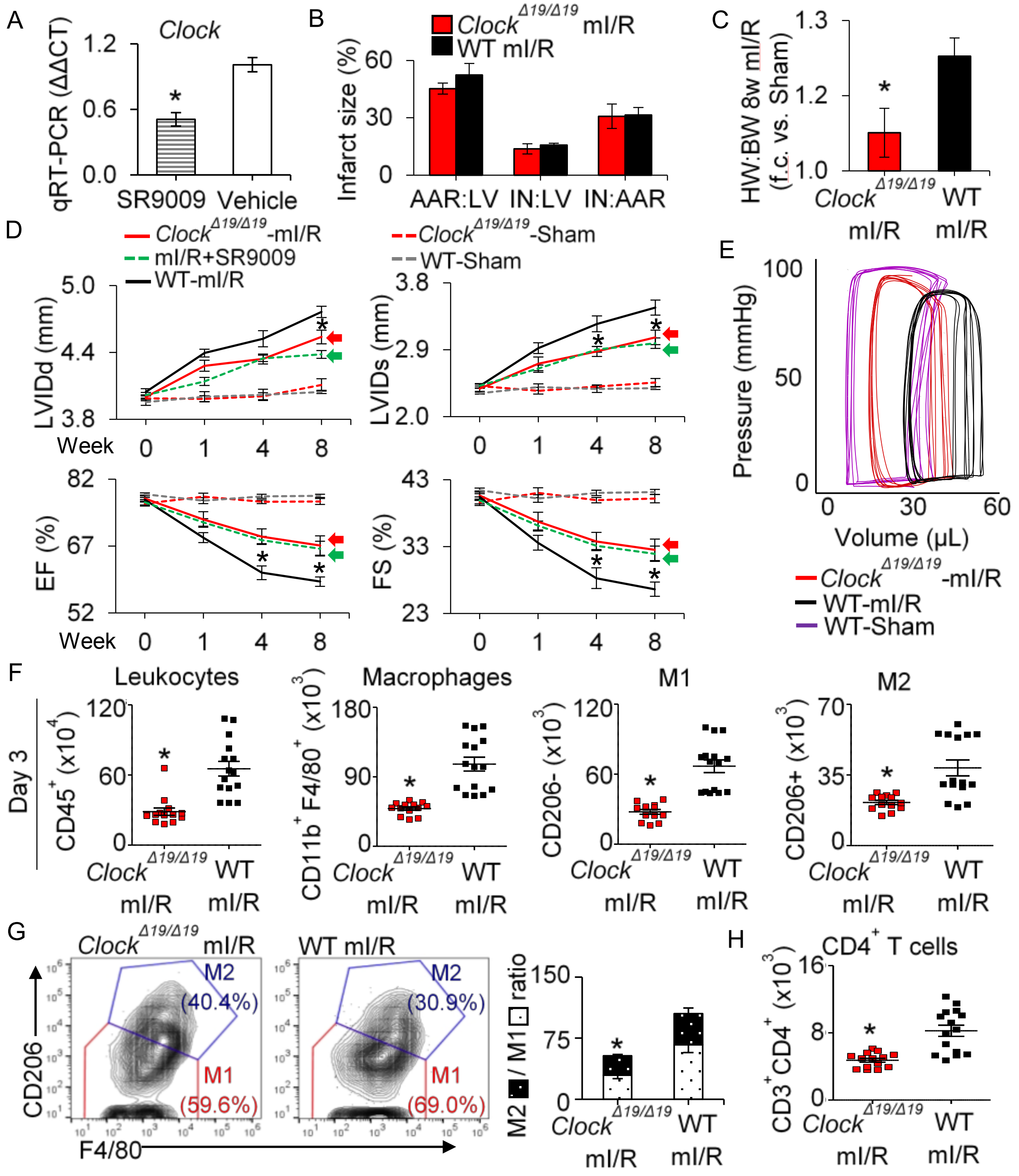

Supplementary Figure 3: *Clock*$^{Δ19/Δ19}$ mI/R mice. (A) SR9009 (ZT06) reduces *Clock* mRNA in WT hearts, consistent with enhanced REV-ERB repressor activity, ZT07, *p<0.001, n=4. (B) Infarcts were similar at day 1 post-mI/R, but (C) *Clock*$^{Δ19/Δ19}$ mice have smaller hearts by 8 weeks post-mI/R vs. WT mI/R. (D) Heart failure outcomes in *Clock*$^{Δ19/Δ19}$ mice (red) are similar to WT mice treated with SR9009 (green), consistent with targeting the circadian mechanism to benefit outcome. Echocardiography, and (E) *in vivo* hemodynamics; see Supplementary Table 4 for values. (F) As compared to WT mI/R mice, *Clock*$^{Δ19/Δ19}$ hearts have fewer leukocytes (CD45$^+$; left) and macrophages (CD11b$^+$F4/80$^+$; middle) in infarcted myocardium, including both M1 (CD206$^-$) and M2 (CD206$^+$) (right) subpopulations, with (G) a greater proportion of reparative M2 cells, and (H) fewer CD4$^+$ T-cells, day 3, * p<0.001, n=5 hearts/group, see Supplementary Table 5 for values.

Supplementary Figure 4

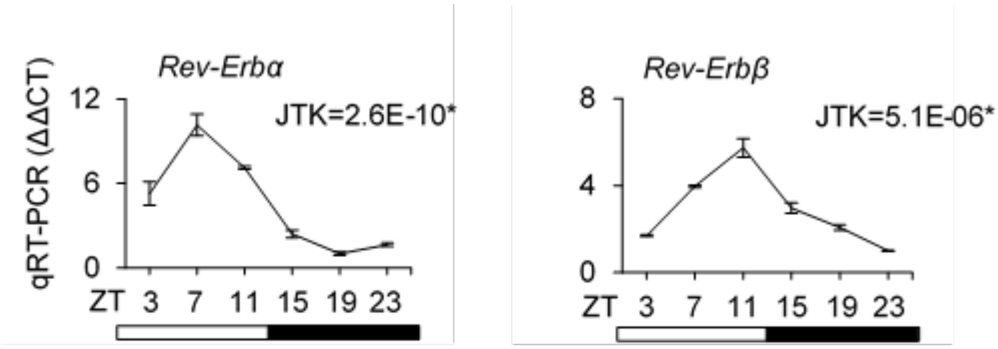

Supplementary Figure 4: Diurnal expression of Rev-Erb in the mouse heart.
Rev-Erbα (left) and Rev-Erbβ (right) cardiac mRNA expression was assessed every 4 hours over 24 hours by RT-PCR. Peak expression was used to determine the timing of SR9009 treatment. JTK_CYCLE is a well-documented method used to estimate period, phase and amplitude, with a threshold of *p<0.05 to identify rhythmicity. n=3 mice per time point, and 2 technical replicates by PCR.

Supplementary Figure 5

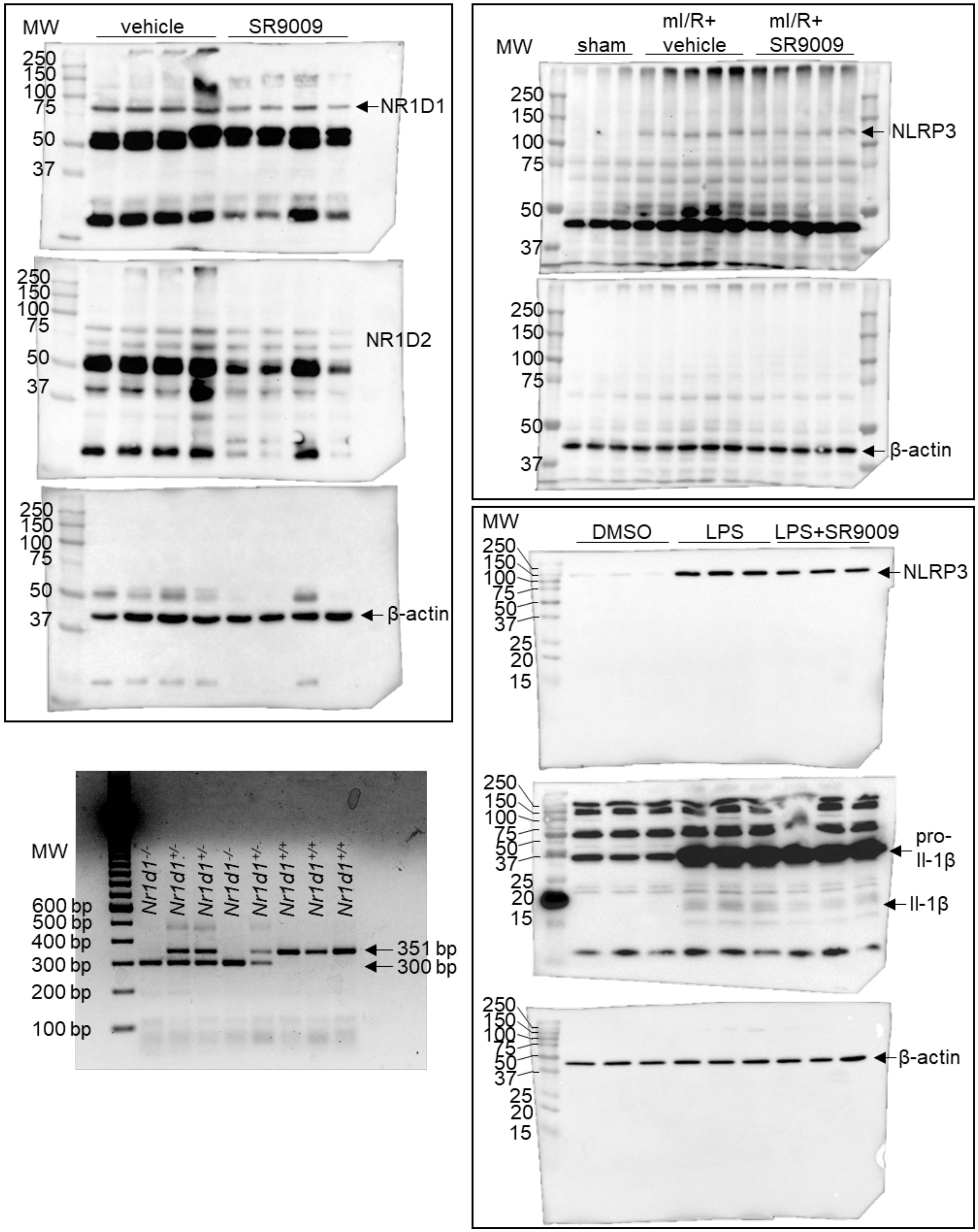

Supplementary Figure 5: Western blots and gels corresponding to Fig. 2b, 3b, 3g, and 4f. Boxes denote reprobed blots.

Supplementary Table 1. Short-term treatment with SR9009 reduces *Rev-Erbα/β* and cytokine mRNA expression in the remodeling heart.

Gene	Day	Sham	mI/R+SR9009	mI/R+Vehicle
n		5	5	5
Rev-Erbα	0	1.01±0.08	-	-
	1		0.31±0.02*	0.39±0.01
	2		0.27±0.01*	0.33±0.02
	3		0.20±0.02*	0.33±0.03
	5		0.38±0.02*	0.58±0.08
	7		0.61±0.06	0.71±0.04
	14		0.90±0.04	0.93±0.02
Rev-Erbβ	0	1.01±0.07	-	-
	1		0.62±0.04*	0.82±0.10
	2		0.70±0.03*	0.84±0.05
	3		0.49±0.07*	0.72±0.09
	5		0.88±0.03*	1.08±0.09
	7		0.93±0.05	1.04±0.07
	14		1.00±0.04	1.02±0.07
Ccl2	0	0.94±0.10	-	-
	1		15.75±0.98*	23.60±2.19
	2		18.92±2.41*	32.15±5.04
	3		17.99±1.85	21.83±6.21
	5		1.88±0.23*	5.17±0.75
	7		3.02±0.23	2.82±0.28
	14		2.06±0.72	1.33±0.11
Il-6	0	0.99±0.17	-	-
	1		13.54±1.15*	25.72±3.62
	2		22.07±2.46*	34.11±5.38
	3		13.22±1.21*	20.02±2.89
	5		9.32±0.34*	16.75±3.44
	7		5.79±0.66	7.16±0.91
	14		3.37±1.23	3.28±0.52
Ccl7	0	1.12±0.15	-	-
	1		3.14±0.35*	5.91±0.63
	2		5.88±0.92*	9.32±1.60
	3		4.61±0.52	6.02±2.06
	5		0.49±0.08*	1.25±0.13
	7		1.03±0.15	0.84±0.06
	14		0.83±0.34	0.41±0.04

*$p<0.05$ mI/R+SR9009 vs. mI/R+vehicle, mean±SEM.

Supplementary Table 2. **Short-term treatment with SR9009 post-mI/R reduces early inflammatory responses.**

	Day 1 post-mI/R			Day 3 post-mI/R	
	**Sham**	**mI/R+SR9009**	**mI/R+Vehicle**	**mI/R+SR9009**	**mI/R+Vehicle**
n	5	5	5	5	5
**Cell Type** ($x10^3$)					
Leukocytes ($CD45^+$)	120±49	759±38	780±123	202±43*	1,042±206
Macrophages ($CD11b^+F4/80^+$)	51±8	56±12*	72±9	40±5*	180±41
M1 macrophages ($CD11b^+F4/80^+$ $CD206^-$)	18±3	42±9*	59±8	22±2*	120±28
M2 macrophages ($CD11b^+F4/80^+$ $CD206^+$)	33±4	10±1*	14±1	20±3*	60±10

Flow cytometry analyses, *p<0.05 mI/R+SR9009 vs. mI/R+vehicle, mean±SEM.

Supplementary Table 3. REV-ERB (Nr1d1) knockout abates SR9009 efficacy post-mI/R.

	***Nr1d1$^{-/-}$* mI/R +SR9009**	***Nr1d1$^{-/-}$* mI/R +Vehicle**	***Nr1d1$^{+/+}$* mI/R +SR9009**	***Nr1d1$^{+/+}$* mI/R +Vehicle**
n	8	8	8	8
**Baseline**				
LVIDd (mm)	3.94±0.01	3.96±0.02	3.95±0.01	3.95±0.02
LVIDs (mm)	2.35±0.02	2.37±0.02	2.38±0.02	2.37±0.03
EF (%)	77.27±0.57	77.18±0.38	76.81±0.41	76.79±0.55
FS (%)	40.25±0.49	40.14±0.34	39.81±0.38	39.85±0.47
HR (bpm)	450±13	450±4	463±8	469±9
BW (g)	22.38±0.70	21.78±0.67	22.84±0.77	23.45±0.44
**1 week post-mI/R**				
LVIDd (mm)	4.34±0.02	4.34±0.04	4.11±0.02*	4.28±0.02
LVIDs (mm)	2.97±0.01	2.95±0.04	2.57±0.03*	2.84±0.03
EF (%)	66.39±0.43	66.09±0.77	73.92±0.80*	69.12±0.55
FS (%)	31.69±0.31	32.07±0.35	36.98±0.80*	33.67±0.40
HR (bpm)	424±11	435±8	450±11	454±12
**4 weeks post-mI/R**				
LVIDd (mm)	4.40±0.03	4.40±0.03	4.15±0.03*	4.33±0.03
LVIDs (mm)	3.11±0.05	3.09±0.04	2.68±0.04*	2.99±0.06
EF (%)	62.99±0.88	63.47±0.74	71.56±0.73*	65.31±1.43
FS (%)	29.39±0.57	29.73±0.50	35.51±0.55*	31.05±0.97
HR (bpm)	424±8	443±8	454±11	462±7

Echocardiography data showing LVIDd, left ventricle internal dimensions at diastole, and LVIDs at systole; %EF, % ejection fraction; %FS, % fractional shortening; HR, heart rate; BW, body weight. *p<0.05, *Nr1d1$^{+/+}$* mI/R +SR9009 vs. *Nr1d1$^{+/+}$* mI/R+vehicle, mean ± SEM.

Supplementary Table 4. ***Clock***$^{\Delta19/\Delta19}$**-mI/R mice have better cardiac outcome vs. WT-mI/R.**

	Clock$^{\Delta19/\Delta19}$ mI/R	WT-mI/R	*Clock*$^{\Delta19/\Delta19}$ Sham	WT-Sham
**Echocardiography**				
**Baseline**				
LVIDd (mm)	4.00±0.02	4.03±0.02	3.98±0.02	3.97±0.02
LVIDs (mm)	2.38±0.04	2.38±0.02	2.40±0.02	2.34±0.03
EF (%)	77.65±0.78	78.03±0.56	76.81±0.45	78.22±0.69
FS (%)	40.62±0.70	40.93±0.52	39.87±0.39	41.08±0.63
HR (bpm)	467±9	463±4	460±9	472±10
**1 week post-mI/R**				
LVIDd (mm)	4.28±0.05	4.40±0.04	3.98±0.03	4.00±0.04
LVIDs (mm)	2.71±0.09	2.92±0.07	2.35±0.05	2.40±0.04
EF (%)	72.97±1.75	68.90±1.51	78.11±0.80	77.18±0.66
FS (%)	36.72±1.41	33.57±1.10	41.05±0.75	40.19±0.57
HR (bpm)	433±10	466±13	438±22	464±3
**4 weeks post-mI/R**				
LVIDd (mm)	4.34±0.04	4.52±0.07	4.01±0.03	4.02±0.05
LVIDs (mm)	2.88±0.07*	3.25±0.11	2.41±0.02	2.37±0.04
EF (%)	69.13±1.77*	61.04±2.36	76.95±0.44	78.19±0.43
FS (%)	33.78±1.37*	28.28±1.55	39.95±0.39	41.04±0.41
HR (bpm)	452±10	461±11	438±6	480±9
**8 weeks post-mI/R**				
LVIDd (mm)	4.54±0.06*	4.76±0.05	4.11±0.06	4.05±0.01
LVIDs (mm)	3.07±0.10*	3.47±0.10	2.46±0.05	2.38±0.02
EF (%)	67.16±2.17*	59.04±2.12	77.04±0.66	78.27±0.38
FS (%)	32.52±1.62*	26.63±1.06	40.18±0.62	41.16±0.37
HR (bpm)	445±9	479±3	461±6	515±14
**Hemodynamics**				
LVESP (mmHg)	93.36±0.72	92.43±0.85	99.97±1.21	99.76±0.58
LVEDP (mmHg)	1.05±0.80	3.74±1.33	0.04±0.76	-0.17±1.02
LVESV (µl)	27.41±2.47*	35.95±1.54	11.13±2.06	12.09±1.25
LVEDV (µl)	46.23±1.11*	54.60±1.94	35.67±2.00	33.04±0.84
CO (mL/min)	11.04±0.27*	9.34±0.42	12.92±0.60	13.81±0.52
HW:BW (mg/g)	4.68±0.21*	5.20±0.15	4.35±0.18	4.24±0.07
HR (bpm)	553±16	504±26	518±16	573±11

LVIDd, left ventricle internal dimensions at diastole; LVIDs, LV internal dimensions at systole; %EF, % ejection fraction; %FS, % fractional shortening; HR, heart rate; LVESP, left ventricle (LV) end systolic pressure; LVEDP, LV end diastolic pressure; LVESV, LV end systolic volume; LVEDV, LV end diastolic volume; CO, cardiac output; HW, heart weight; BW, body weight. n=5 mice/group, *$p<0.05$ *Clock*$^{\Delta19/\Delta19}$-mI/R vs. WT-mI/R, mean±SEM.

Supplementary Table 5. **Less inflammation in *Clock*$^{\Delta19/\Delta19}$-mI/R vs. WT-mI/R hearts.**

	***Clock*$^{\Delta19/\Delta19}$-mI/R**	**WT-mI/R**
n	5	5
**Cell Type (x10^3)**		
Leukocytes ($CD45^+$)	290±35*	653±116
Macrophages ($CD11b^+F4/80^+$)	49±4*	106±17
M1 macrophages ($CD11b^+F4/80^+CD206^-$)	30±4*	67±9
M2 macrophages ($CD11b^+F4/80^+CD206^+$)	24±2*	38±7
M1:M (%)	55±3*	64±3
M2:M (%)	46±2*	35±3
M1:M2	1±0*	2±0
T cells ($CD3^+CD4^+$)	5±0*	8±1

Flow cytometry at day 3 post-mI/R. *p<0.05, mean±SEM.

Supplementary Table 6. **Time-of-day dosing (chronotherapy) with SR9009.**

	mI/R+SR9009 (ZT06) a	mI/R+SR9009 (ZT18/06) b	mI/R+SR9009 (ZT18) c	mI/R+Vehicle (ZT18) d
n	7	7	7	7
**Echocardiography**				
**Baseline**				
LVIDd (mm)	4.02±0.03	4.01±0.01	4.00±0.03	3.99±0.02
LVIDs (mm)	2.42±0.03	2.40±0.01	2.37±0.03	2.40±0.03
EF (%)	76.81±0.53	77.11±0.32	77.90±0.46	76.93±0.63
FS (%)	39.92±0.47	40.14±0.23	40.80±0.43	39.95±0.56
HR (bpm)	471±6	479±8	462±9	479±4
**1 week post-mI/R**				
LVIDd (mm)	4.14±0.02*	4.23±0.06*	4.35±0.04	4.46±0.06
LVIDs (mm)	2.63±0.03*	2.82±0.09*	2.97±0.05	3.15±0.08
EF (%)	72.88±0.83*	68.73±1.99*	65.99±1.24	63.14±1.55
FS (%)	36.56±0.69*	33.50±1.35*	31.84±0.80	29.56±0.97
HR (bpm)	463±7	444±15	433±6	426±16
**4 weeks post-mI/R**				
LVIDd (mm)	4.32±0.03*	4.39±0.03*	4.42±0.03	4.50±0.04
LVIDs (mm)	2.89±0.05*	3.01±0.05*	3.14±0.05	3.29±0.05
EF (%)	68.05±1.15*	65.95±1.21*	62.19±1.60	58.86±0.96
FS (%)	32.88±0.80*	31.43±0.82*	28.91±1.06	26.79±0.59
HR (bpm)	470±6	468±10	430±5	437±14
**8 weeks post-mI/R**				
LVIDd (mm)	4.36±0.04*	4.52±0.06*	4.69±0.03	4.74±0.07
LVIDs (mm)	2.94±0.07*	3.11±0.09*	3.42±0.02	3.57±0.10
EF (%)	67.63±1.79*	65.58±1.70*	59.12±0.26	55.48±1.89
FS (%)	32.71±1.27*	31.25±1.10*	26.90±0.16	24.83±1.05
HR (bpm)	469±9	471±12	432±8	432±8

LVIDd, left ventricle internal dimensions at diastole; LVIDs, LV internal dimensions at systole; % EF, % ejection fraction; % FS, % fractional shortening; HR, heart rate. [a]mI/R at sleep time (ZT06) and SR9009 at reperfusion (ZT06) then ZT06 for 5 days. [b]mI/R at wake time (ZT18) and SR9009 at reperfusion (ZT18) then ZT06 for 5 days. [c]mI/R at wake time (ZT18) and SR9009 at reperfusion (ZT18) then ZT18 for 5 days. [d]mI/R at wake time (ZT18) and vehicle control. *$p<0.05$, mI/R+SR9009 (ZT18/06) vs. mI/R+vehicle, mean±SEM.

Supplementary Table 7. RT-PCR primers.

Gene	**Sequence 5'-3'**
Clock$^{\Delta 19/\Delta 19}$	TGGGGTAAAAAGACCTCTTGCC<br>GGTCAAGGGCTACAGTTCG<br>AGCACCTTCCTTTGCAGTTCG<br>TGTGCTCAGACAGAATAAGTA
Nr1d1$^{-/-}$	CCACAGCTCTTAGCCCAGAC<br>CCTTCTATCGCCTTCTTGACG<br>CTTCCCACTCCAGTCACCAC
Rev-Erbα	GGGCACAAGCAACATTACCA<br>CACGTCCCCACACACCTTAC
Rev-Erbβ	GCCCAAAGATGAGCATTC<br>CTCTATCTGGCTGATGTC
Clock	GCCTCAGCAGCAACAGCAGC<br>ACCGCATGCCAACTGAGCGA
Il-6	GCTAAGGACCAAGACCATCCAAT<br>GGCATAACGCACTAGGTTTGC
Ccl2	GTCCCTGTCATGCTTCTGG<br>TCTTGCTGGTGAATGAGTAGC
Ccl7	TCTCTCACTCTCTTTCTCCAC<br>GGATCTTTTGTTTCTTGACATAGC
Nlrp3	CATGTTGCCTGTTCTTCCAGAC<br>CGGTTGGTGCTTAGACTTGAGA
Il-1β	TGGGCCTCAAAGGAAAGAAT<br>TGGGTATTGCTTGGGATCCA
Il-18	TCCAGCATCAGGACAAAG<br>ACGCAAGAGTCTTCTGAC
Histone	GCAAGAGTGCGCCCTCTACTG<br>GGCCTCACTTGCCTCCTGCAA
