# Supplementary material for: SR9009 administered for one day after myocardial ischemia-reperfusion prevents heart failure in mice by targeting the cardiac inflammasome
Source: Commun Biol. 2019 Oct 3;2:353. doi: 10.1038/s42003-019-0595-z (PMC6776554; doi:10.1038/s42003-019-0595-z)
Supplement: Supplementary file 2 — Description of Additional Supplementary Files [file 42003_2019_595_MOESM2_ESM.docx]

**Description of Additional Supplementary Files**

**File Name:** Supplementary Data 1

**Description:** Source data file
