# Supplementary material for: SR9009 administered for one day after myocardial ischemia-reperfusion prevents heart failure in mice by targeting the cardiac inflammasome
Source: Commun Biol. 2019 Oct 3;2:353. doi: 10.1038/s42003-019-0595-z (PMC6776554; doi:10.1038/s42003-019-0595-z)
Supplement: Supplementary file 4 — Reporting Summary [file 42003_2019_595_MOESM4_ESM.pdf]

Dr. Tami Martino

Corresponding author(s): DBPR_COMMSBIO-18-1452_T

Last updated by author(s): Aug 13, 2019

nature research

Reporting Summary

Nature Research wishes to improve the reproducibility of the work that we publish. This form provides structure for consistency and transparency in reporting. For further information on Nature Research policies, see Authors & Referees and the Editorial Policy Checklist .

Statistics

For all statistical analyses, confirm that the following items are present in the figure legend, table legend, main text, or Methods section.

n/a	Confirmed	
☐	☒	The exact sample size (*n*) for each experimental group/condition, given as a discrete number and unit of measurement
☐	☒	A statement on whether measurements were taken from distinct samples or whether the same sample was measured repeatedly
☐	☒	The statistical test(s) used AND whether they are one- or two-sided<br>*Only common tests should be described solely by name; describe more complex techniques in the Methods section.*
☒	☐	A description of all covariates tested
☐	☒	A description of any assumptions or corrections, such as tests of normality and adjustment for multiple comparisons
☐	☒	A full description of the statistical parameters including central tendency (e.g. means) or other basic estimates (e.g. regression coefficient) AND variation (e.g. standard deviation) or associated estimates of uncertainty (e.g. confidence intervals)
☒	☐	For null hypothesis testing, the test statistic (e.g. *F*, *t*, *r*) with confidence intervals, effect sizes, degrees of freedom and *P* value noted<br>*Give P values as exact values whenever suitable.*
☒	☐	For Bayesian analysis, information on the choice of priors and Markov chain Monte Carlo settings
☒	☐	For hierarchical and complex designs, identification of the appropriate level for tests and full reporting of outcomes
☒	☐	Estimates of effect sizes (e.g. Cohen's *d*, Pearson's *r*), indicating how they were calculated

Our web collection on statistics for biologists contains articles on many of the points above.

Software and code

Policy information about availability of computer code

Data collection	No software was used
Data analysis	Excel, GraphPad Prism version 8.1.2, JTK_CYCLE v3.0

For manuscripts utilizing custom algorithms or software that are central to the research but not yet described in published literature, software must be made available to editors/reviewers. We strongly encourage code deposition in a community repository (e.g. GitHub). See the Nature Research guidelines for submitting code & software for further information.

Data

Policy information about availability of data

All manuscripts must include a data availability statement. This statement should provide the following information, where applicable:

- Accession codes, unique identifiers, or web links for publicly available datasets
- A list of figures that have associated raw data
- A description of any restrictions on data availability

All data and materials are available from the corresponding author upon request. Raw data from Figs. 2-5 are shown in Supplementary Information.

Field-specific reporting

Please select the one below that is the best fit for your research. If you are not sure, read the appropriate sections before making your selection.

☒ Life sciences ☐ Behavioural & social sciences ☐ Ecological, evolutionary & environmental sciences

For a reference copy of the document with all sections, see nature.com/documents/nr-reporting-summary-flat.pdf

Life sciences study design

All studies must disclose on these points even when the disclosure is negative.

Sample size	Sample sizes are based on previous published data using pathophysiological techniques of echocardiography, hemodynamics, etc. All approaches are documented.
Data exclusions	No data are excluded.
Replication	All experiments were done using technical and biological replicates, and data were confirmed using a variety of genetic and pharmacologic approaches.
Randomization	All animals were randomized in the studies.
Blinding	All data evaluations were done blinded by the analyzer.

Reporting for specific materials, systems and methods

We require information from authors about some types of materials, experimental systems and methods used in many studies. Here, indicate whether each material, system or method listed is relevant to your study. If you are not sure if a list item applies to your research, read the appropriate section before selecting a response.

Materials & experimental systems

n/a	Involved in the study	
☐	☒	Antibodies
☒	☐	Eukaryotic cell lines
☒	☐	Palaeontology
☐	☒	Animals and other organisms
☒	☐	Human research participants
☒	☐	Clinical data

Methods

n/a	Involved in the study	
☒	☐	ChIP-seq
☐	☒	Flow cytometry
☒	☐	MRI-based neuroimaging

Antibodies

Antibodies used	anti-CD16/32 (clone 93, eBioscience), anti-CD45-APC (clone 30-F11, eBioscience), anti-CD11b-PE (clone M1/70, Biolegend), anti-F4/80-FITC (clone BM8, Biolegend), anti-CD206-AlexaFluor647 (clone C068C2, Biolegend), anti-CD3e-FITC (clone 145-2C11, Biolegend), anti-CD4-PE (clone GK1.5, Biolegend), anti-Ki67-APC (clone 16A8, Biolegend), anti-CD45.1-APC (cloneA20, Biolegend), anti-CD45.2-PE (clone 104, Biolegend), REV-ERBα (mouse monoclonal, clone 4F6; 1:1000; Abgent), REV-ERBβ (mouse monoclonal, clone D-8; 1:1000; Santa Cruz Biotechnology), NLRP3 (mouse monoclonal, clone Cryo-2; 1:1000; Adipogen), IL-1β (rabbit polyclonal, GTX74034; 1:1000; GeneTex), and β-actin (mouse monoclonal, clone C4; 1:10,000; Millipore)
Validation	Each commercially available antibody was validated for the species and application by the vendor. No non-commercial antibodies were used.

Animals and other organisms

Policy information about studies involving animals; ARRIVE guidelines recommended for reporting animal research

Laboratory animals	Male C57BL/6 mice from Charles River, male ClockΔ19/Δ19 mutant, and male Rev-Erb (Nr1d1-/-) knockout mice (on C57Bl/6 background) as documented in text.
Wild animals	The study did not involve wild animals.
Field-collected samples	The study did not involve samples collected from the field.
Ethics oversight	All animal work was conducted under the guidelines of the Canadian Council on Animal Care, with AUPs for all experiments approved by the University of Guelph Institutional Animal Care and Use Committee.

Note that full information on the approval of the study protocol must also be provided in the manuscript.

Flow Cytometry

Plots

Confirm that:

☒ The axis labels state the marker and fluorochrome used (e.g. CD4-FITC).

☒ The axis scales are clearly visible. Include numbers along axes only for bottom left plot of group (a 'group' is an analysis of identical markers).

☒ All plots are contour plots with outliers or pseudocolor plots.

☒ A numerical value for number of cells or percentage (with statistics) is provided.

Methodology

Sample preparation	A separate set of hearts was collected to quantify immune cell recruitment to infarcted myocardium post-m/IR. Briefly, animals were sacrificed by isoflurane and cervical dislocation. Hearts were exposed, perfused via cardiac puncture with 100 units heparin in 5 mL saline, and then removed and rinsed in saline. Epicardial fat, atria and the free wall of the right ventricle were removed and the left ventricle was minced into 1-2 mm pieces with a razor blade. Hearts were digested for 60 min at 37 degrees Celcius with collagenase type II (1 mg/mL; Worthington Biochemical Corporation) and DNase I (60 U/mL; Roche Diagnostics Corporation) in 10 mL RPMI 1640 media (Life Technologies). Released cells were separated using a 70μm cell strainer (Fisher Scientific). Cell count and viability (>90%) were determined using the trypan blue exclusion method. Aliquots were diluted to 1x10^6 cells in 50μl staining buffer (PBS, 2% BSA) and incubated with anti-CD16/32 (clone 93, eBioscience) for 5 min at 4 degrees Celcius to block Fcγ receptors. After blocking, cell suspensions were incubated with fluorochrome-conjugated antibodies in 50 μl staining buffer in the dark for 30 min at 4 degrees Celcius. To examine immunocyte recruitment cells were stained for total leukocytes with anti-CD45-APC (clone 30-F11, eBioscience), or for macrophages with anti-CD11b-PE (clone M1/70, Biolegend) and anti-F4/80-FITC (clone BM8, Biolegend) and anti-CD206-AlexaFluor647 (clone C068C2, Biolegend; M1=206-, M2=206+), or for T-cells with anti-CD3e-FITC (clone 145-2C11, Biolegend) and anti-CD4-PE (clone GK1.5, Biolegend), or anti-Ki67-APC (clone 16A8, Biolegend).
Instrument	Samples were analyzed on an Accuri C6 Flow Cytometer (BD Biosciences).
Software	BD Accuri C6 Software (BD Biosciences) and FlowJo software (Tree Star).
Cell population abundance	For flow cytometry analysis, a minimum of 10,000 events per sample were recorded in triplicate.
Gating strategy	The gating strategy selected for single cells, with the removal of debris and cell doublets. Single cells were then gated as live leukocytes determined on the basis of FSC/SSC characteristics. From this population, CD45+ cells were selected as the leukocyte population. Macrophages were identified as CD11b+ and F4/80+ cells, based on single-stained and fluorescence-minus-one controls. F4/80+CD206+ cells were identified as M2 macrophages and the CD206- population as M1 macrophages. T cells were identified as CD45+CD3+CD4+ cells on the basis of gating determined by single-stained and fluorescence-minus-one controls. A representative example of the gating strategy is shown in Supplementary Figure 1.

☒ Tick this box to confirm that a figure exemplifying the gating strategy is provided in the Supplementary Information.
